# Optimizing drought tolerance in cassava through genomic selection

**DOI:** 10.3389/fpls.2024.1483340

**Published:** 2024-12-16

**Authors:** Weverton Gomes da Costa, Massaine Bandeira e Souza, Camila Ferreira Azevedo, Moyses Nascimento, Carolina Vianna Morgante, Jerônimo Constantino Borel, Eder Jorge de Oliveira

**Affiliations:** ^1^ Laboratório de Inteligência Computacional e Aprendizado Estatístico - LICAE, Departamento de Estatística, Universidade Federal de Viçosa, Viçosa, Minas Gerais, Brazil; ^2^ Nugene, Embrapa Mandioca e Fruticultura, Cruz das Almas, Bahia, Brazil; ^3^ Embrapa Semiárido, Petrolina, Pernambuco, Brazil; ^4^ Departamento de Agronomia, Universidade Federal do Vale do São Francisco, Petrolina, Pernambuco, Brazil

**Keywords:** *Manihot esculenta* Crantz, mixed model, breeding, genotype selection, genomic values

## Abstract

The complexity of selecting for drought tolerance in cassava, influenced by multiple factors, demands innovative approaches to plant selection. This study aimed to identify cassava clones with tolerance to water stress by employing truncated selection and selection based on genomic values for population improvement and genotype evaluation *per se*. The Best Linear Unbiased Predictions (BLUPs), Genomic Estimated Breeding Values (GEBVs), and Genomic Estimated Genotypic Values (GETGVs) were obtained based on different prediction models via genomic selection. The selection intensity ranged from 10 to 30%. A wide range of BLUPs for agronomic traits indicate desirable genetic variability for initiating genomic selection cycles to improve cassava’s drought tolerance. SNP-based heritability (*h* ^2^) and broad-sense heritabilities (*H* ^2^) under water deficit were low magnitude (<0.40) for 8 to 12 agronomic traits evaluated. Genomic predictive abilities were below the levels of phenotypic heritability, varying by trait and prediction model, with the lowest and highest predictive abilities observed for starch content (0.15 – 0.22) and root length (0.34 – 0.36). Some agronomic traits of greater importance, such as fresh root yield (0.29 – 0.31) and shoot yield (0.31 – 0.32), showed good predictive ability, while dry matter content had lower predictive ability (0.16 – 0.22). The G-BLUP and RKHS methods presented higher predictive abilities, suggesting that incorporating kinship effects can be beneficial, especially in challenging environments. The selection differential based on a 15% selection intensity (62 genotypes) was higher for economically significant traits, such as starch content, shoot yield, and fresh root yield, both for population improvement (GEBVs) and for evaluating genotype’s performance per (GETGVs). The lower costs of genotyping offer advantages over conventional phenotyping, making genomic selection a promising approach to increasing genetic gains for drought tolerance in cassava and reducing the breeding cycle to at least half the conventional time.

## 1 Introduction

On a global scale, cassava (*Manihot esculenta* Crantz) plays a crucial role in both food security and energy production, serving as a primary source of income and carbohydrates for millions of people in tropical regions (Kayondo et al., 2018; de Oliveira et al., 2021; Silva et al., 2021). In Brazil, where cassava is extensively cultivated, even under adverse conditions of water and nutrient availability (de Andrade et al., 2019; Wei et al., 2020), climate change and water stress pose substantial challenges to its successful production.

In this scenario, finding effective breeding strategies becomes essential, especially when dealing with the complexity of the drought tolerance — a key factor that negatively affects cassava growth and productivity (El-Sharkawy, 2004; Vitor et al., 2019). When plants face water shortages, they undergo changes at morphological, physiological, biochemical, and molecular levels, which ultimately reduce growth and yield. Drought tolerance is a multifaceted trait controlled by numerous genes, transcription factors, miRNAs, hormones, proteins, cofactors, ions, and metabolites (OkogBenin et al., 2013; Budak et al., 2015). Thus, assessing genetic variability in cassava is essential for unlocking the crop’s full potential, particularly in semi-arid regions, by identifying and selecting genotypes that show greater resilience to water scarcity (Silva et al., 2021).

While traditional methods of selecting clones based on phenotypic traits and best linear unbiased predictors (BLUPs) are still valuable, their main limitation lies in the long generation intervals. This makes it clear that faster, more agile strategies are needed. Truncation selection, which ranks individuals based on their phenotypic traits and uses the top performers for crossing, can also fall short when the populations doesn’t offer enough variation across all relevant traits (Sampaio Filho et al., 2023). Moreover, the complex factors involved in drought response have made it difficult to develop drought-tolerant cultivars through conventional breeding techniques (Mohammadi, 2018).

Cassava, due to its clonal propagation and heterozygous nature, faces notable challenges in implementing truncated selection strategies due to the impact of intrafamilial genetic variations. These variations can significantly affect the accuracy of identifying superior genotypes. Additionally, the expected performance of progeny may differ from BLUP-based predictions for parents, largely due to the presence of non-additive effects for economically important traits in cassava (Wolfe et al., 2021).

Given these challenges, innovative breeding strategies are crucial. Genomic selection (GS), which uses genetic markers spread across the genome to predict genomic estimated breeding values (GEBVs), offers considerable potential to accelerate genetic progress in targeted populations. By improving selection accuracy, GS helps identify the most promising clones playing a vital role in selecting new parents for crosses (Meuwissen et al., 2001; Hayes et al., 2009). GS enables the early selection and recombination of promising genotypes without the need for direct phenotypic evaluation. This approach is particularly valuable in situations where phenotypic selection is costly or inefficient, such as in the seedling stage of cassava, where the heritability of important agronomic traits is very low (de Andrade et al., 2019).

In parallel with population improvement based on GEBV, selecting clones through traditional breeding pipeline using multi-environment field tests can identify superior clones for release as varieties. In these cases, selection should be based on the estimated total genomic breeding value (GETGV) of an individual, which includes non-additive genetic effects such as dominance (Wolfe et al., 2021).

The combination of BLUPs and genomic selection holds promise for reducing generation intervals and improving selection accuracy during early developmental stages (Werner et al., 2023). Therefore, it is important to develop and assess selection strategies that that align with both specific and broader goals of cassava breeding programs, especially in water-stressed environments. This study aims to assess the efficacy of genomic selection methods, including additive-dominant G-BLUP, alongside traditional selection based on BLUPs, GEBVs, and GETGVs of clones. By integrating these approaches, the study seeks to reduce generation intervals and enhance selection accuracy, particularly in early development stages, providing crucial insights for addressing water stress challenges in cassava cultivation.

## 2 Materials and methods

### 2.1 Phenotypic data collection

Experiments were conducted at two locations in the state of Pernambuco, Brazil: the Campus of Agricultural Sciences of the Universidade Federal do Vale do São Francisco in Petrolina (9°19’16.1”S 40°32’32.4”W, altitude 373 m) and the Bebedouro Experimental Station of Embrapa Semiárido, also in Petrolina (9°05’49.2”S 40°18’24.1”W, altitude 376 m). The climate and environmental data for two locations in Petrolina, Brazil, spanning a five-year period, are provided in **Supplementary Table S1**. A total of 446 cassava genotypes were evaluated, including local varieties and improved varieties known for drought tolerance, which were either harvested from semi-arid regions or selected under drought conditions. Evaluations were conducted over four seasons, from 2016 to 2020 (**Table 1**).

**Table 1:** Year of evaluation and number of genotypes assessed. The diagonal entries indicate the number of genotypes evaluated in that specific year, while the off-diagonal entries indicate overlapping genotypes assessed in different years.

Year	2017	2018	2019	2020
2017	165	42	22	14
2018	42	138	39	16
2019	22	39	133	29
2020	14	16	29	138

The experimental design was a randomized complete block with four replications. Each plot consisted of ten plants (two rows of five plants) spaced 0.90 m apart between rows and 0.80 m apart between plants. For planting, stem cuttings of 16-18 cm in length were used, following the standard agricultural practices recommended for the crop, as described in Souza et al. (2006). All experiments were irrigated for up to three months after planting (MAP). Water was supplied every two days via inline dripping (4 L h^-1^) based on the plants’ evapotranspiration, estimated using data from a nearby meteorological station. After this period, irrigation was suspended until harvest to assess drought tolerance in the cassava genotypes.

According to Vitor et al. (2019), by 4 months after planting, the yield potential of cassava genotypes is almost fully determined, with minimal changes in the ranking of genotypes for various agronomic traits when compared to harvests at 12 months after planting (end of the cycle). Therefore, in our study, the comparative evaluations of cassava genotypes for drought tolerance were conducted at 6 MAP to enable the highest possible number of phenotypic assessments in a shorter period of time. The main traits evaluated during harvest included: 1) Fresh root yield (FRY), representing the total weight of all roots in the plot, converted to tons per hectare (t ha^-1^); 2) Shoot yield (ShY), representing the weight of the aboveground parts of all plants in the plot, including stems, leaves, and petioles, also converted to tons per hectare (t ha^-1^); 3) Dry matter content of roots (DMC), determined as a percentage using the gravimetric method (Kawano et al., 1987); 4) Number of stems per plant (Nstem.Plant); 5) Harvest index (HI), the ratio of fresh root weight to total biomass, including both aboveground and belowground parts of the plants, expressed as a percentage (%); 6) Plant height (Plant.Height), measured from the soil level to the plant meristem using a graduated scale, expressed in meters (m); 7) Starch content (StC), obtained by specific weight according to Kawano et al. (1987); 8) Number of roots per plant (N_Roots); 9) Starch yield (StY), obtained by multiplying starch content by fresh root yield, expressed in tons per hectare (t ha^-1^); 10) Root Length (Root.Le), measured the length of the root from the tip to the base in cm; 11) Root Diameter (Root.Di): measured the diameter of the root at its midpoint using a digital caliper, recorded in cm; and 12) Stem Diameter (Stem.D): measured the diameter of the stem at ground level using a digital caliper, recorded in cm.

### 2.2 Phenotypic analyses

A linear mixed model was employed to estimate BLUPs through the analysis of multi-environmental trials. Prior to the analysis, a preliminary descriptive analysis of the data was conducted to detect and remove any highly discrepant values. BLUPs were obtained by fitting a multi-environmental model (with year as the environment) to the following linear mixed model: $y_{ijklm}=µ+E_{i}+b_{k\left(i\right)}+R_{l}+C_{m}+G_{j}+GE_{ij}+e_{ijk},$ where $y_{ijk}\;$ is the phenotype value of the j^th^ genotype in the k^th^ block and i^th^ environment, µ is the overall mean, $E_{i}$ is the random effect of the i^th^ environment, $b_{k\left(i\right)}$ is the fixed effect of the k^th^ block within the i^th^ environment, $R_{l}$ is the random effect of the l^th^ row, $C_{m}$ is the random effect of the m^th^ column, $G_{j}$ is the random effect of the j^th^ genotype, $GE_{ij}$ is the random effect of the j^th^ genotype in the i^th^ environment, and $e_{ijk}\;$ is the random error ( $e_{ijk}\sim N\left(0\sigma {,}_{r}^{2}\right),$ where and $\sigma_{r}^{2}$ represents the residual variance). The random vectors follow the distributions: $E\sim N\left(0,I\sigma_{e}^{2}\right)$ , $R\sim N\left(0,I\sigma_{rw}^{2}\right)$ , $C\sim N\left(0,I\sigma_{c}^{2}\right)$ , $G\sim N\left(0,I\sigma_{g}^{2}\right)$ , $GE\sim N\left(0,I\sigma_{ge}^{2}\right)$ . Here, $\sigma_{g}^{2}$ represents genetic variance, $\sigma_{\text{e}}^{2}$ represents environmental variance, $\sigma_{\text{rw}}^{2}$ represents row variance, $\sigma_{\text{c}}^{2}$ represents column variance $\sigma_{ge}^{2}$ represents the variance due to the interaction between genotype and environment. The residual distribution was thoroughly examined, along with its relationship to the fitted values, to assess the adequacy of the model and identify any potential patterns or deviations.

SNP-based heritability (*h* ^2^) and broad-sense heritabilitiy (*H* ^2^) were also calculated to evaluate the contribution of genetic and environmental factors to the phenotypic variation of the evaluated assessed, using the formula: $h^{2}=\frac{\sigma_{A}^{2}}{\sigma_{A}^{2}+\sigma_{R}^{2}}$ and $H^{2}=\frac{\sigma_{g}^{2}}{\sigma_{g}^{2}+\sigma_{r}^{2}}$ , where $\sigma_{A}^{2}$ and $\sigma_{R}^{2}$ represent the additive and residual components based on markers, and $\sigma_{g}^{2}$ $\sigma_{r}^{2}$ represent the genetic and residual components based on phenotype. The sommer package (Covarrubias-Pazaran, 2016) in R software version 4.2.3 (R Core Team, 2023) was used to obtain the BLUPs and variance components.

### 2.3 Genotyping and SNP quality control

Genomic DNA was extracted using the cetyltrimethylammonium bromide (CTAB) protocol described by Doyle and Doyle (1987). Subsequently, DNA samples were sent to the Genomic Diversity Facility at Cornell University (http://www.biotech.cornell.edu/brc/genomic-diversity-facility) for Genotyping by Sequencing (GBS) as described by Hamblin and Rabbi (2014). A comprehensive set of 27,045 single nucleotide polymorphisms (SNPs) distributed across all 18 cassava chromosomes was obtained.

To ensure the reliability of the data, quality control was performed on the genotypic information. Markers with minimum allele frequencies (MAF) below 0.01 were systematically excluded from further analyses. After this quality control step, the marker matrix was refined to include 22,779 high-quality SNPs, which were subsequently used in subsequent analyses. The GBS dataset generated as part of this study is publicly available through Cassavabase (https://www.cassavabase.org/).

### 2.4 Genomic selection

This study evaluated various genomic selection methods, including RR-BLUP, G-BLUP (both with additive and dominance effects), RKHS, BayesA, BayesB, and Random Forest, each based on distinct statistical assumptions. The RR-BLUP model is expressed as: $y_{d}=1\mu +Mu+є$ , where $y_{d}\;$ is the vector of BLUPs from the phenotypic analysis, μ is the overall mean, 1 is a vector with elements equal to 1, *u* is the marker effects vector, ***M*** is the marker matrix, and є is the vector of residual effects. The random vectors follow the distributions: $u\;\sim \;N\left(0,I\sigma_{u}^{2}\right)$ , and $є\;\sim \;N\left(0,I\sigma_{e}^{2}\right)$ , where $\sigma_{u_{a}}^{2}$ is the additive marker variance, and $\sigma_{e}^{2}$ is the residual variance.

The additive-dominant genetic model of G-BLUP is expressed as: $y_{d}=1\mu +Za+Hd+є$ , where $y_{d}\;$ is the vector of BLUPs; μ is the overall mean; 1 is a vector with elements equal to 1, ***a*** is the vector of random additive effects of individuals, ***d*** is the vector of random dominant effects of individuals, and є is the vector of residual effects. ***Z*** is the incidence matrix for genetic effects ***a*** and ***d***. The random vectors follow the distributions $a\;\sim \;N\left(0,\;G\sigma_{a}^{2}\right)$ , $d\;\sim \;N\left(0,D\sigma_{d}^{2}\right)$ and $є\;\sim \;N\left(0,I\sigma_{e}^{2}\right)$ , where $\sigma_{a}^{2}$ is the additive variance, $\sigma_{d}^{2}$ is the dominant variance and ${\;}_{e}^{2}$ is the residual variance. The additive relationship matrix is calculated as: $G\;=\;\frac{ZZ'\;}{2\sum^{}p_{i}\left(1\;-\;p_{i}\right)}$ and the dominant relationship matrix used is that described by Vitezica et al. (2013): $\;D\;=\frac{HH'\;}{2\sum^{}p_{i}q_{i}\left(1\;-\;p_{i}q_{i}\right)}\;$ .

The RKHS method model is given by: $y_{d}=1\mu +Zg+є$ , where $y_{d}\;$ is the vector of BLUPs, *μ* is the overall mean, 1 is a vector with elements equal to 1, ***g*** is the vector of random genotypic effects with $g\;\sim \;N\left(0,K\sigma_{g}^{2}\right)$ where ${\;}_{g}^{2}$ is the genetic variance, є is the vector of residual effects with $є\;\sim \;N\left(0,I\sigma_{e}^{2}\right)$ , ${\;}_{e}^{2}$ is the residual variance. **Z** is the incidence matrices of ***g*** and ***K*** is a Gaussian matrix estimated by $K\;=\;exp\left(\frac{-hDist}{median\left(Dist\right)}\right)$ , where ℎ is the reduction coefficient for the ***K*** values, and Dist is the Euclidean distance of the coded marker matrix ***M*** (Gianola et al., 2006; Crossa et al., 2010).

For the Bayes A and Bayes B methods, the same model as RR-BLUP is used. For BayesA, the prior distributions for the i-th marker effects are $u_{a_{i}}|\sigma {\;}_{a_{i}}^{2}\sim N\left(0,\sigma {\;}_{a_{i}}^{2}\right)$ and $u_{d_{i}}|\sigma {\;}_{d_{i}}^{2}\sim N\left(0,\sigma {\;}_{d_{i}}^{2}\right)$ . For BayesB, the prior distributions are $u_{a_{i}}|\sigma {\;}_{a_{i}}^{2},\pi \sim \left(1-\pi \right)N\left(0,\sigma {\;}_{a_{i}}^{2}=0\right)+\pi N\left(0,\sigma {\;}_{a_{i}}^{2}\right)$ and $u_{d_{i}}|\sigma {\;}_{d_{i}}^{2},\pi \sim \left(1-\pi \right)N\left(0,\sigma {\;}_{d_{i}}^{2}=0\right)+\pi N\left(0,\sigma {\;}_{d_{i}}^{2}\right).$ $\sigma_{u_{a}}^{2}$ is the additive marker variance and $\sigma_{u_{d}}^{2}$ is the dominant marker variance. The variances assumed to follow an inverted chi-square distribution scaled, and π follows a beta distribution. The Gibbs sampler with 20,000 iterations was used to fit the model, discarding the first 5,000 samples as burn-in and saving one in every ten samples to calculate posterior means of the parameters.

The Random Forest (RF) method is an extension of regression tree, designed to improve prediction accuracy by generating multiple models from bootstrapped samples of data. Each tree is built using a random subset of predictors, aiming to identify the optimal partition that creates homogeneous groups within the data. RF enhances prediction by averaging the outputs of several trees, which reduces overfitting and boosts generalization ability (Prasad et al., 2006).

The RF algorithm uses recursive binary splitting to select the best predictor, $X_{j}$ which is a marker *j*. It evaluates the split $\left\{x|x_{j}<s\right\}$ e $\left\{x|x_{j}\geq s\right\}$ to minimize the residual sum of squares (RSS), represented as:


$$R_{1}\left(j,\;s\right)=\;\left\{X|X_{j}\;<\;s\right\}e\;R_{2}\left(j,\;s\right)=\;\left\{X|X_{j}\;\geq \;s\right\},$$

The goal is to find the values of *j* and *s* that minimize the following equation:


$$\sum_{i:x_{i}\in R_{1}\left(j,s\right)}{\left(y_{i}-{\hat{y}}_{R_{1}}\right)}^{2}+\sum_{i:x_{i}\in R_{2}\left(j,s\right)}{\left(y_{i}-{\hat{y}}_{R_{2}}\right)}^{2}$$

where ${\hat{y}}_{R_{1}}$ is the mean response variable of the training observations in region $R_{1}\left(j,\;s\right)$ , ${\hat{y}}_{R_{2}}$ is the mean response variable in region $R_{2}\left(j,\;s\right)$ , and $y_{i}$ is the true value of the response for each individual observation (James et al., 2021).

In RF, a total of B models are generated, denoted as ${\hat{f}}^{1}\left(x\right),\;{\hat{f}}^{2}\left(x\right),\ldots ,\;{\hat{f}}^{B}\left(x\right)$ . A key feature of RF is that each tree is built using a random subset of predictors at each node, promoting diversity among the trees and enhancing the overall model’s robustness (Boehmke and Greenwell, 2019). In this study, the RF model was implemented using the RandomForest package (Liaw and Wiener, 2002). The model was set to generate 500 trees, and at each split, the number of variables randomly sampled as candidates was set to p/3, where *p* represents the total number of markers (Prasad et al., 2006).

We employed 5-fold cross-validation with five repetitions to estimate predictive ability using the formula: $r_{\hat{y}y}=cor\left({\hat{y}}_{val},y_{val}\right)$ , where ${\hat{y}}_{val}$ is the genomic estimated breeding values (GEBVs) or genomic estimated genotypic values (GETGVs) of the validation population from each method, and $y_{val}$ is the BLUPs of the validation population.

The sommer package (Covarrubias-Pazaran, 2016) was used for fitting the RR-BLUP, G-BLUP and RKHS models, while the BGLR package (Pérez and De Los Campos, 2014) was used for Bayes A and Bayes B models. All analyses were performed using R software 4.2.3 (R Core Team, 2023).

### 2.5 Clone selection

For the first cycle of genomic selection focused on drought tolerance, we used a combined approach to select GS-C0 clones for the crossing block, incorporating both BLUPs and GEBVs/GETGVs. GEBVs predict average performance in random matings, suitable for recurrent selection, while GETGVs assess individual clone performance, ideal for cultivar advancement. Combining these methods aimed to balance gains across multiple traits and improve population performance. Clones were ranked using a selection index that weighted traits according to their importance.

The clones were ranked based on the following selection index: *InS*=(5×*Nstem*.*Plant*) + (5×*Stem*.*D*) + (5×*Root*.*Di*) + (5×*Root*.*Le*) + (5×*Plant*.*Height*) + (10×*StC*) + (10×*HI*) + (10×*StY*) + (10×*ShY*) + (15×*DMC*) + (15×*NRoots*) + (20×*FRY*), where *InS* represents the value of the individual selection index for each clone. The coefficients associated with each trait reflect their respective weights within the selection index. Each trait corresponds to the values of GEBV or GETGV.

In the context of selecting clones for population improvement and identifying promising clones for future crosses, selection intensities ranging from 10% to 30% were explored for each evaluated trait. Two distinct strategies were implemented: selection based on GEBVs and GETGVs.

The Kappa coefficient (Cohen, 1960), was used to evaluate agreement between the selection methods in identifying superior clones. The primary goal was to compare the effectiveness of these methods in identifying high-performing clones for future crosses, ultimately optimizing the formation of an improved cassava population.

## 3 Results

### 3.1 Variance components and estimation of genetic parameters

The distributions of BLUPs for each trait are shown in **Supplementary Figure S1**. Although distinct distribution patterns were observed for agronomic traits, all exhibited variability in their BLUPs. This broad genetic variability is highly desirable in the training population (GS-C0) as it is important for initiating genomic selection cycles aimed at improving drought tolerance in cassava. Additionally, the residual distribution and its relationship with the fitted values were analyzed to assess the model’s goodness of fit (**Supplementary Figures S2, S3**). The analysis revealed that the residuals largely adhere to a normal distribution and demonstrate homogeneity of variance across the spectrum of fitted values.

**Figure 1:**
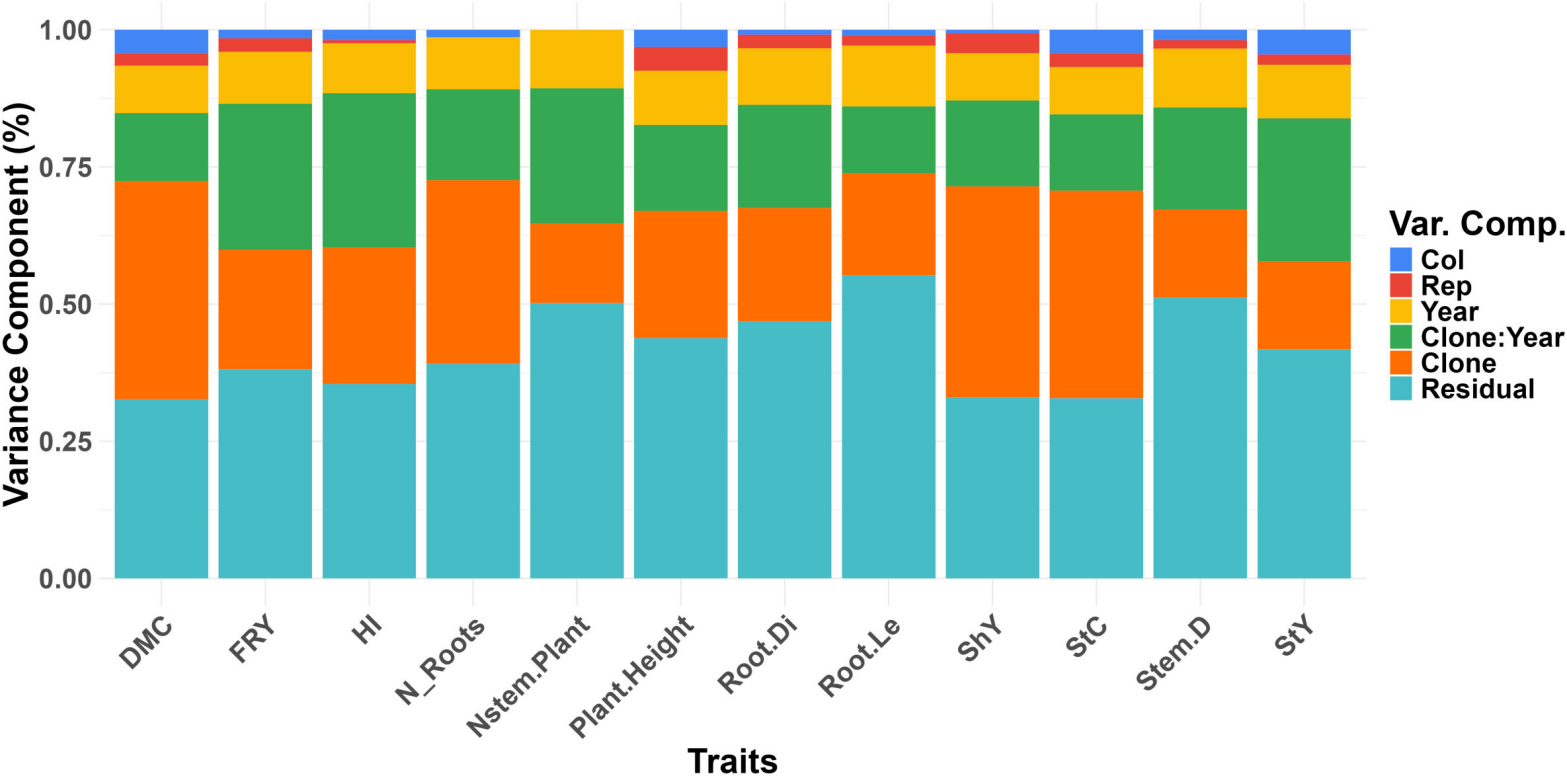
Distribution of variance components of the traits evaluated in field trials under water deficit for the traits: dry matter content (DMC), fresh root yield (FRY), harvest index (HI), number of roots per plant (N_Roots), number of stems per plant (Nstem.Plant), plant height (Plant.Height), root diameter (Root.Di), root length (Root.Le), shoot yield (ShY), starch content (StC), stem diameter (Stem.D) and starch yield (StY).

Estimates of variance components derived from the joint analysis across the four environments provided a thorough understanding of the factors contributing to phenotypic variation in the evaluated traits (**Figure 1**). Both residual and genotypic effects, as well as their interaction, were significant (**Supplementary Table S2**). Residual effects accounted for the largest portion of variation in all traits, ranging from 32.99% for ShY to 55.21% for Root.Le. Traits like Root.Le, Stem.D, and Nstem.Plant showed the highest residual variation, with more than 50% of the variation explained by residuals. In contrast, genetic variances for traits such as ShY, StC, and DMC were relatively high, though still comparable to the residual variances (~40% each). The genetic variation of the Nstem.Plant trait was considerably lower, representing just 14.52% of the total variation. It is noteworthy that the effect of the evaluation year was nearly constant for all traits, ranging from 8.57% to 11.05%. However, the effect of the year × clone interaction was more pronounced for traits like FRY, HI, Nstem.Plant, and StY, although it remained similar in magnitude to the genetic variance for these traits. The analysis showed that row and column effects, although statistically significant in some cases, contributed only small portions of the overall variation. For example, the row effect for shoot yield (ShY) accounted for just 0.64%, while the column effect was slightly higher at 1.37%. For dry matter content (DMC), the row and column effects were 4.27% and 4.51%, respectively. These results highlight that while row and column effects are detectable, their contributions to phenotypic variation are minimal compared to the dominant residual and genetic effects, reinforcing the primary role of genetic and residual factors in shaping the traits studied.

The results reveal that traits with higher residual effects, such as Root.Le, Stem.D, Root.Di, and Nstem.Plant, exhibited lower broad-sense heritability (*H* ^2^ ranging from 0.224 to 0.279) (**Table 2**). For the remaining traits, *H* ^2^ estimates were above 0.30, suggesting that, while environmental factors contribute to phenotypic expression, genetic inheritance is still significant. ShY exhibited the highest *H* ^2^ value (0.523), highlighting the high influence of genetic factors on this trait’s expression. Traits such as N_Roots, DMC, and StC displayed moderate *H* ^2^ values, indicating good potential for improvement through genetic selection.

**Table 2:** Broad-sense heritability (*H* ^2^), SNP-based heritability (*h* ^2^), mean and range for several cassava agronomic traits evaluated in field trials under water deficit.

Trait	*h* ^2^	*H* ^2^	Mean/range
Number of roots per plant	0.199	0.462	29.06 (11.98 - 48.34)
Fresh root yield	0.410	0.363	4.95 (0.12 - 22.2)
Shoot yield	0.397	0.538	24.56 (1.57 - 71.97)
Dry matter content	0.309	0.549	4.29 (0.12 - 15.67)
Starch yield	0.440	0.277	2.13 (1.00 - 6.67)
Plant height	0.385	0.346	1.19 (0.36 - 3.03)
Harvest index	0.316	0.413	28.88 (6.12 - 63.3)
Starch content	0.312	0.535	23.21 (7.00 - 47.33)
Root length	0.351	0.253	14.23 (0.69 - 61.17)
Root diameter	0.294	0.306	24.42 (7.33 - 43.69)
Stem diameter	0.450	0.239	1.52 (0.02 - 8.87)
Number of stems per plant	0.251	0.225	2.11 (1.01 - 4.37)

SNP-based heritability (*h* ^2^), which capture additive genetic variance and can help estimate the narrow-sense heritability of traits, revealed a significant influence of genetic factors on the observed phenotypic variation, ranging between 0.199 and 0.450. Among these, StY and Stem.D stands out with the highest SNP-based heritability (>0.40), suggesting that most of the variation in root production is attributable to genetic factors. Other traits with medium magnitude of *h* ^2^ include ShY (0.397), FRY (0.410), and Plant.Height (0.385). For traits such as N_Roots, StC, DMC, and ShY the *H* ^2^ estimate was higher than *h* ^2^, while for Stem.D, the opposite was observed.

### 3.2 Predictive performance of different genomic selection methods

**Figure 2:**
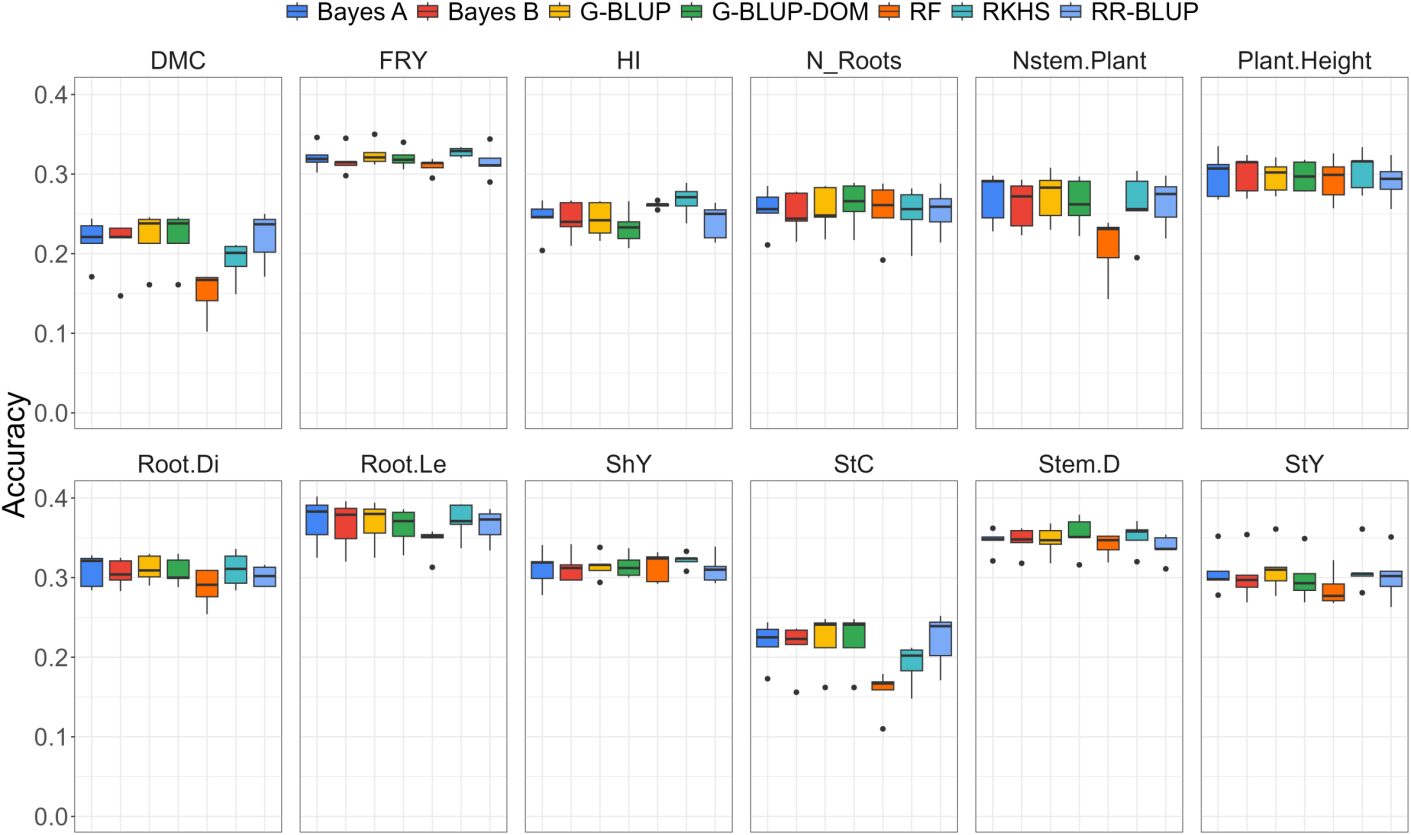
Boxplot of predictive ability for different genomic selection methods, including Bayes (A), Bayes (B), G-BLUP additive (G-BLUP), G-BLUP additive-dominant (G-BLUP-DOM), Random Forest (RF), Reproducing Kernel Hilbert Space (RKHS), and RR-BLUP for various traits evaluated under water deficit, such as dry matter content (DMC), fresh root yield (FRY), harvest index (HI), number of roots per plant (N_Roots), number of stems per plant (Nstem.Plant), plant height (Plant.Height), root diameter (Root.Di), root length (Root.Le), shoot yield (ShY), starch content (StC), stem diameter (Stem.D) and starch yield (StY).

A comprehensive examination of genomic prediction methods revealed distinct patterns in predictive ability for various agronomic traits under water deficit conditions. Overall, all genomic ability estimates fell below phenotypic heritability levels (**Figure 2**; **Table 2**), indicating an inherent challenge in achieving high predictive ability under water stress conditions.

In the case of cassava, our study revealed prediction abilities ranging from 0.150 to 0.371, depending on the trait and the model used. For DMC, prediction abilities ranged from 0.150 to 0.221, with the RR-BLUP model being the most accurate. For traits such as FRY and HI, the prediction abilities ranged from 0.310 to 0.328 and 0.233 to 0.267, respectively, with the RKHS model providing the highest predictive ability. For the N_Roots, predictive abilities ranged from 0.250 to 0.262, with the G-BLUP-DOM model being the most accurate. Prediction abilities for other traits, such as Nstem.Plant(0.208 to 0.272), Plant.Height(0.280 to 0.304), Root.Di(0.288 to 0.311), and Root.Le(0.345 to 0.371), were also fairly similar, with the RKHS model showing the highest predictive ability. Similarly, the prediction abilities for traits such as ShY (0.311 to 0.324), StC (0.157 to 0.222), StY (0.286 to 0.311), and Stem.D (0.345 to 0.353) were comparable, though the G-BLUP model achieved the best predictive performance for these traits.

Results indicated that the predictive abilities of different genomic selection methods were generally similar, except for random forest. The random forest method exhibited the lowest predictive ability values across all analyzed traits (**Figure 2**), particularly for traits such as DMC, Nstem.Plant, and StC. This finding suggests a limitation in the effective application of random forest for predicting agronomic traits under water stress conditions, highlighting the need to explore more robust alternatives.

In contrast, the G-BLUP and G-BLUP-DOM methods showed higher predictive ability estimates with minimal variations between them. The RKHS method also demonstrated high predictive abilities for several traits, including FRY, DMC, and HI. These results suggest that incorporating kinship effects may confer an advantage in prediction ability, especially in challenging environments. However, it is essential to note that overall, predictive ability did not exceed 0.40 for any trait, indicating a general limitation in accurately predicting traits under water stress. Notably, traits such as DMC, StC, and HI exhibited the lowest average predictive ability, highlighting specific complexities in modeling and predicting these attributes under water deficit conditions.

In summary, selecting the genomic prediction method for water stress data should involve careful consideration of the traits of interest, taking into account the stability and consistency of the method across different agronomic contexts. Given their superior predictive abilities for most evaluated agronomic traits, we focused our further analyses solely on the G-BLUP and G-BLUP-DOM methods.

The results indicate that the predictive abilities of various genomic selection methods were generally comparable, with the exception of the random forest method. Random forest exhibited the lowest predictive ability across all analyzed traits (**Figure 2**), particularly for traits such as DMC, Nstem.Plant, and StC. This suggests that random forest may have limitations in effectively predicting agronomic traits under water stress conditions, underscoring the need to explore more robust alternatives.

A thorough examination of genomic prediction methods revealed distinct patterns in predictive ability for different agronomic traits under water deficit conditions. Overall, the genomic prediction abilities were lower than the phenotypic heritability levels (**Figure 2**; **Table 2**), highlighting the inherent challenge of achieving high predictive accuracy under water stress.

### 3.3 Comparison between selection methods

**Figure 3:**
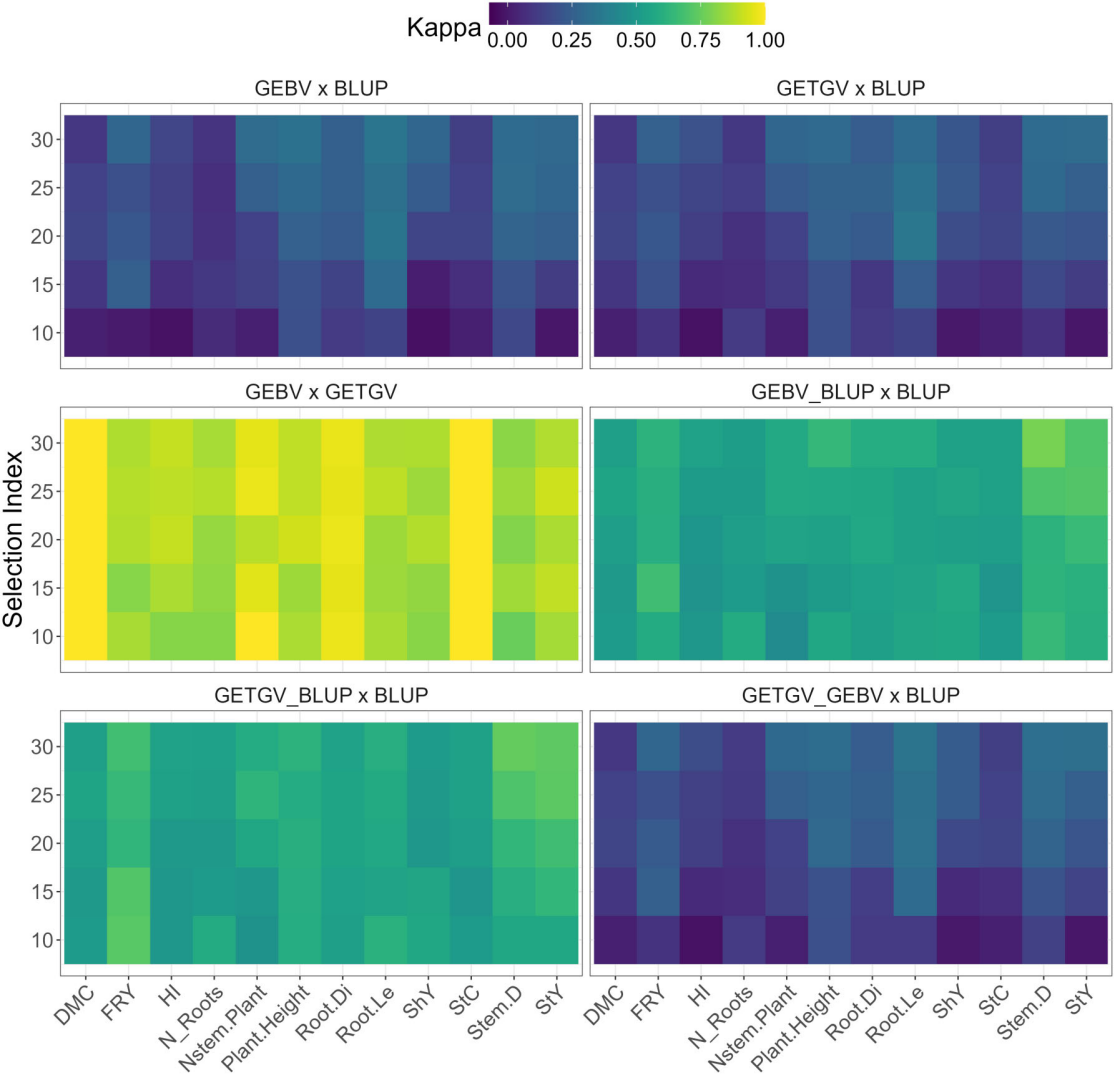
Cohen’s Kappa of coincidence in selecting cassava clones based on its high GEBVs and BLUPs considering different selection proportion (10% to 30%—SP) for several traits, such as: starch yield (StY), stem diameter (Stem.D), starch content (StC), shoot yield (ShY), root length (Root.Le), root diameter (Root.Di), plant height (Plant.Height), number of stems per plant (Nstem.Plant), number of roots per plant (N_Roots), harvest index (HI), fresh root yield (FRY), and dry matter content (DMC).

The comparative analysis encompassed truncated selection based on BLUP and genomic selection based on GEBVs and GETGVs obtained using the additive G-BLUP and additive-dominant G-BLUP methods, respectively. Different levels of selection index (SI), ranging from 10% to 30%, were explored using the Kappa coefficient as the evaluation metric (**Figure 3**; **Supplementary Table S3**).

When individually evaluating the genomic selection methods compared to the BLUP selection method (GEBV × BLUP and GETGV × BLUP), the Kappa coefficients remained below 0.4 (**Figure 3**), regardless of the selection index (SI) level ranging between 10% and 30%. This suggests a limited agreement between the genotypes selected by truncation selection with BLUP and genomic selection based on GEBV or GETGV. Similar results were observed when employing combined selection between GETGV and GEBV compared to truncated selection (GETGV_GEBV × BLUP). The trait with the lowest concordance between selection approaches was HI, followed by N_Roots and StC. Conversely, for traits Root.Le and Plant.Height, there was a higher degree of agreement in the selection of cassava genotypes based on different approaches.

In the comparative analysis between genomic methods (GEBV × GETGV), notable consistency in selection was observed due to the high agreement between genotypes selected through these approaches, especially for traits DMC, StC, Nstem.Plant, and Root.Di, with coefficients equal to or close to 1.00 at any selection intensity. Therefore, even if there were statistical differences in the predictive ability of the additive and additive-dominant G-BLUP methods, the expected genetic gains would be very similar. A similar situation was also observed when using combined selection of GEBV_BLUP and GETGV_BLUP compared to selection based solely on BLUP. In these cases, the concordance coefficients between methods ranged from 0.44 to 0.78.

### 3.4 Selection of clones for population improvement and agronomic performance

**Figure 4:**
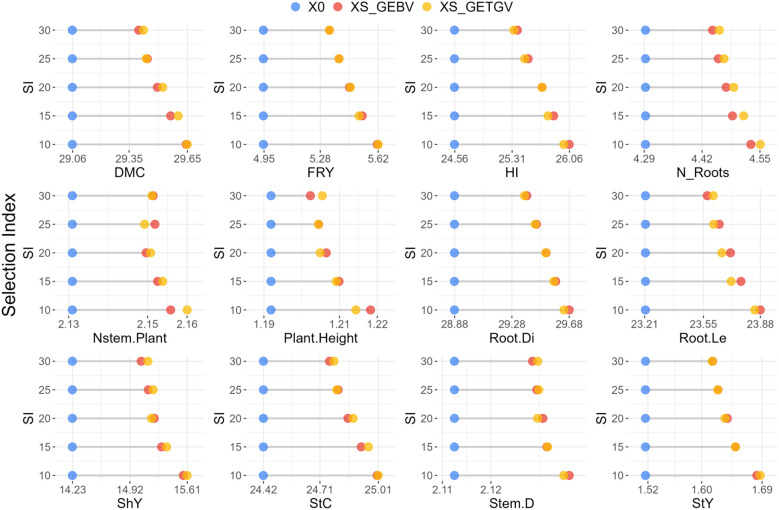
Average of the evaluated cassava population under water deficit conditions (X0) alongside the average of the improved population (XS) based on the GEBVs and GETGVs of cassava genotypes. This analysis considers selection proportions ranging from 10% to 30% of the original population for various agronomic traits. Dry matter content (DMC), fresh root yield (FRY), harvest index (HI), number of roots per plant (N_Roots), number of stems per plant (Nstem.Plant), plant height (Plant.Height), root diameter (Root.Di), root length (Root.Le), shoot yield (ShY), starch content (StC), stem diameter (Stem.D) and starch yield (StY).

The GEBVs and GETGVs were used to select cassava clones for population improvement and to confirm agronomic performance per se for recommendation as new cultivars in the target regions of the project, respectively (**Figure 4**; **Supplementary Table S4**). The averages of the selected population (XS) and the selection differentials (SD) based on GEBV and GETGV were computed for each trait and selection intensity, ranging from 10 to 30%. At the highest selection intensity (10%), the selection differentials of cassava clones to be used as parents were higher for traits such as FRY (13.5%), StY 10.9%), and ShY (9.4%), while traits such as Stem.D, Nstem,Plant had selection differentials below 2% compared to the average of the original population (**Figure 4**; **Supplementary Table S4**). This trend was also observed in the selection of clones based on GETGV, except that the Plant.Height trait exhibited a selection differential above 2% compared to the average of the evaluated population.

Increasing the selection intensity from 10% to 30% led to a reduction in the selection differential of cassava clones for both the average GEBV and GETGV. However, the difference in selection differentials between the 10% and 15% intensities of the original population was relatively low (<20%) for most evaluated traits, except for Plant.Height and FRY (**Figure 4**; **Supplementary Table S4**). On the other hand, the maximum selection intensity (30%) resulted in a reduction in the selection differential compared to the lowest selection intensity (10%), with reductions exceeding 40% for traits such as DMC, FRY, Plant.Height, HI, Root.Le, StC and StY.

Overall, there was a tendency for similar gains from selection based on genomic parameters (GEBV and GETGV), although selection focusing on agronomic performance *per se* based on GETGV resulted in slightly higher gains than selection of parents based on GEBV for most traits (**Figure 4**). Exceptions to this trend were observed for traits DMC, Nstem.Plant and StC at a 25% selection intensity, and for HI, Plant.Height, Root.Di, and Root.Le at almost all levels of selection intensity, where the average GEBV was higher than the GETGV.

## 4 Discussion

### 4.1 Genetic parameters for agronomic traits in cassava under drought tolerance

Our study identified significant interaction effects between environmental factors (year) and genotype (clone) for various cassava traits, particularly for FRY, HI, Nstem.Plant, and StY (**Supplementary Table S2**). Residual effects were notably higher for traits such as Nstem.Plant, Plant.Height, Root.Di, Root.Le, and Stem.D (**Figure 1**). As a result, broad-sense heritability (*H* ^2^) estimates were generally of moderate to low magnitude, ranging from 0.22 to 0.52. These heritability values align with those reported for cassava in typical cultivation environments (rainfed planting with rainfall only during the initial growth stages), such as the findings of de Andrade et al. (2019) for FRY and DMC (0.337 and 0.545, respectively) and Sampaio Filho et al. (2023) for FRY, ShY, DMC, PH, and HI, with *H* ^2^ values of 0.32, 0.30, 0.57, 0.50, and 0.40, respectively.

Another type of heritability that was also found to have a moderate to low magnitude (ranging from 0.201 to 0.44) is SNP-based heritability (*h* ^2^). This represents the portion of trait variation explained by SNPs and is useful for understanding the genetic control over a trait. In many cases, *H* ^2^ and *h* ^2^ were similar, as seen in traits like Nstem.Plant, Root.Di, Plant.Height, FRY, and ShY. However, for certain traits such as N_Roots, StC, DMC, and ShY, *h* ^2^ estimates were lower than *H* ^2^. In general, *h* ^2^ can be lower than *H* ^2^ for several reasons. First, *h* ^2^ typically only captures additive genetic variance, whereas *H* ^2^ includes all genetic effects, including additive, dominance, and epistasis. If non-additive genetic effects are significant for a trait, *H* ^2^ may be higher. Another potential explanation includes: i) incomplete coverage of genetic variance where SNP markers may not capture all genetic variation, especially if the causal variants are rare or not well-represented, and ii) population structure, in which *h* ^2^ estimates can be lower in populations with complex structures or high relatedness because the model may not fully account for all genetic relationships, leading to less variance explained by the SNPs. Although studies on cassava are limited, a recent study by Aghogho et al. (2024) reported lower *h* ^2^ than *H* ^2^ for several root quality traits, such as gari yield, peel loss, and bulk density.

Water deficit directly affects plant growth and physiological development, presenting significant challenges for selecting plants based on their responses to drought stress. Under drought conditions, heritability values for cassava can vary considerably, primarily due to the stress environment. For instance, heritability estimates significantly lower than those observed in this study have been reported for traits like N_roots (*H* ^2^ = 0.25 – de Oliveira et al., 2015) and ShY (*H* ^2^ = 0.26 – Silva et al., 2021), while other traits, such as Root.Di, have shown much higher heritabilities (*H* ^2^ = 0.46 – Vieira et al., 2024). These low heritability estimates pose significant challenges for breeding programs. In crops like wheat and barley, for example, low heritability for productive traits has limited the effectiveness of marker-assisted selection (MAS) and the identification of robust QTLs, complicating the selection of superior genotypes (Isidro et al., 2015; Liu et al., 2018). Similarly, in maize, the low heritability of yield-related traits under drought stress requires multiple selection cycles to achieve meaningful genetic gains, which extends the duration and cost of breeding programs (Lorenzana and Bernardo, 2009).

In breeding for drought tolerance, plant selection has traditionally focused on identifying genotypes that maintain high yield under stress conditions (Sallam et al., 2019; de Oliveira et al., 2021). However, intrinsic factors linked to drought-prone environments, such as limited genetic variation for specific traits, strong genotype × environment interactions, and low heritability, can limit the effectiveness of breeding efforts (Varshney et al., 2021). These factors can complicate the selection of optimal genotypes across different years and growing conditions.

To overcome the challenges posed by low heritability, integrating advanced techniques like genomic selection has become essential for optimizing genetic gains. Genomic selection leverages genetic marker information across the genome to predict the genetic potential of individuals, improving selection accuracy even for traits with low heritability (Heffner et al., 2009). Moreover, conducting multi-environment phenotypic evaluations and exploring the full spectrum of available genetic diversity, including wild relatives, are critical strategies for capturing true genetic variation and improving the efficiency of breeding programs in the medium to long term (Hickey et al., 2014). These approaches help mitigate the impact of low heritability and can lead to significant advancements in crop improvement.

Recent studies have shown that the prediction accuracy of genomic selection for drought tolerance traits often compares favorably with phenotypic heritability values. For instance, in maize, prediction abilities ranged from 0.5 to 0.7, while phenotypic heritability for these traits ranged from 0.4 to 0.6 (Zhang et al., 2017). Similarly, in sorghum, prediction accuracies for grain yield and stay-green traits under drought stress were around 0.6, exceeding the phenotypic heritability values, which ranged from 0.3 to 0.5 (Santantonio and Robbins, 2020). In our study, prediction abilities for various agronomic traits in cassava were lower than their *H* ^2^ estimates. For example, prediction abilities for DMC ranged from 0.150 to 0.221, while *H* ^2^ was 0.549. For FRY, prediction abilities ranged from 0.310 to 0.328, compared to *H* ^2^ of 0.363. Similarly, for HI, prediction abilities ranged from 0.233 to 0.267, with *H* ^2^ ranging from 0.413.

### 4.2 Genomic selection to improve drought tolerance in cassava

Lower genetic gains under drought conditions compared to favorable environments represent significant limitations for breeding programs (Mohammadi, 2018). Overcoming this challenge requires a comprehensive approach that integrates plant breeding, genomics, statistics, experimental design, and strategies for managing genetic diversity. While traditional phenotypic BLUP selection has been, and continues to be, highly valuable, incorporating genomic values (GEBVs or GETGVs) provides a more targeted and efficient strategy, especially in the context of drought tolerance. This is particularly relevant given the high and long-term costs associated with phenotyping cassava populations for drought tolerance.

The predictive performance of genomic selection models is influenced by several factors, including SNP marker density, the number of QTLs, trait heritability and complexity, the genomic selection model, and other factors (Daetwyler et al., 2008; Rosero et al., 2020; da Costa et al., 2022; Yan et al., 2022). In our study on cassava, prediction abilities ranged from 0.154 to 0.371, with traits such as FRY, Plant.Height, Root.Di, Root.Le, ShY, and Stem.D showing at least one prediction model with predictive abilities above 0.30.

In standard cassava cultivation trials (without drought stress), higher prediction accuracies have been reported for traits such as ShY (ranging from 0.72 to 0.77), FRY (ranging from 0.66 to 0.76), and DMC (ranging from 0.67 to 0.68) (Oliveira et al., 2012; Wolfe et al., 2017; Torres et al., 2019). A more recent study by Andrade et al. (2022) reported lower prediction accuracies for FRY (0.48) and DMC (0.57), although these were still higher than the values reported in our study under drought conditions. Literature suggests that predictive accuracies for yield traits under drought stress are generally low in other crops as well. For example, in maize, prediction accuracies for grain yield ranged from low (0.03) to moderate (0.51), even when including dominance effects in the model and using different cross-validation schemes (Dias et al., 2018). Similarly, in wheat, prediction accuracies ranged from 0.33 to 0.67 for productive traits under drought stress (Mohammadi, 2018).

Different genomic prediction models can yield varying prediction abilities due to their different assumptions and characteristics. For instance, the RR-BLUP model assumes that all markers have small, equal effects, which may not be realistic for all traits and can limit its predictive ability when few major-effect QTLs are involved (Clark and van der Werf, 2013; Haile et al., 2021). In contrast, the G-BLUP model uses a genomic relationship matrix to capture genetic variation and is generally robust, though it can still be affected by linkage disequilibrium and population structure (Haile et al., 2021). More advanced models, such as BayesA and BayesB, assume different distributions for marker effects, allowing for more flexible and potentially more accurate modeling of traits controlled by a few large-effect QTLs (Daetwyler et al., 2013; de los Campos et al., 2013; Rosero et al., 2020). Additionally, methods like RKHS and deep learning models can capture complex nonlinear relationships between markers and traits, offering an advantage when such relationships are significant (Gianola et al., 2006; Gota and Gianola, 2014). Since GBLUP is the most commonly implemented prediction model (Nascimento et al., 2024), its results were used as the benchmark in this study.

In this study, while the differences between genomic selection methods were similar in terms of predictive ability, the additive G-BLUP, additive-dominant G-BLUP, and RKHS methods showed slight superiority over other methods for most traits. Due to the advantages of clonal propagation, family structure is often overlooked by breeders in clonal selection (Ceballos et al., 2015), with minimal use of pedigree information. Therefore, genomic selection methods that utilize a genetic relationship matrix, such as G-BLUP and RKHS, can help increase selection gains by using covariance information between individuals to estimate GEBVs (de Andrade et al., 2019).

Other studies have also highlighted the effectiveness of the G-BLUP method for routine genomic prediction in cassava roots (Wolfe et al., 2017; de Andrade et al., 2019). Its main advantage lies in the use of the genetic relationship matrix, which leverages covariance information between individuals to estimate GEBVs. This enables for the estimation of relationships through markers, even without prior knowledge of relatedness, potentially improving selection gains (Meuwissen et al., 2001). G-BLUP assumes an infinitesimal genetic architecture, with nearly equal and small contributions from all genomic regions to phenotypes, which contrasts from Bayesian methods that allow for variance to vary between SNP loci and emphasize main effect loci and variable selection (Crossa et al., 2010; Wolfe et al., 2017). Although methods like RKHS and RF can model epistasis (Kayondo et al., 2018), G-BLUP is still preferred due to its reduced computational demands and simplicity (Hernandez et al., 2020) especially when compared to more complex parametric methods like Bayesian Alphabet (Gianola et al., 2009).

While Barreto et al. (2024) suggested that modeling both additive and dominant effects, depending on the environment, could improve genomic prediction in drought-stressed trials, our results showed that prediction accuracy for most traits was relatively low under drought conditions and early harvests (6 months after planting). Additive effects predominated for most traits, as evidenced by high correlations between GEBVs and GETGVs. Other studies have shown that traits such as dry matter content and root diameter are primarily controlled by additive effects, while traits like fresh root yield, root number, harvest index, and plant height tend to be influenced by non-additive effects (Zacarias and Labuschagne, 2010; Tumuhimbise et al., 2014; Wolfe et al., 2016; Varona et al., 2018). However, when the dominance effect accounts for a minor portion of genetic variance, as observed in this study, using both additive and additive-dominant models to understand trait control can improve predictions of total genetic effects (Dias et al., 2018; Barreto et al., 2024).

### 4.3 Selection of cassava clones for recombination and evaluation of agronomic performance

According to El-Sharkawy (2004), traditional methods for selecting parental lines to enhance drought tolerance and adaptation to infertile soils have resulted in the development of improved cassava cultivars with high yields in favorable environments and stability under stress. However, achieving progressively higher genetic gains for various traits has become increasingly challenging for conventional breeding programs. As a result, new approaches, such as genomic selection can aid in predicting agronomic performance and identifying the best lines and parental combinations for population improvement or cultivar validation (Mohammadi, 2018).

In cassava population improvement, the cycle time for reintroducing a clone into a new breeding cycle is lengthy due to several biological constraints, including low flowering rates, long breeding cycles, limited genetic diversity, and low rates of planting material multiplication (Kayondo et al., 2018). Genomic selection can optimize and expedite the breeding pipeline by reducing the time needed for improvement through the selection of superior parental genotypes at the seedling stage based solely on genotyping data (Heffner et al., 2009; Wolfe et al., 2017).

Typically, a selection cycle in cassava requires one to two years to produce botanical seeds from the clones to be tested, followed by an additional two to four years for field evaluations (Oliveira et al., 2012; Wolfe et al., 2017; Torres et al., 2019). Selection decisions are made throughout this process to reduce the number of genotypes evaluated in replicated trials across multiple locations (Torres et al., 2019). The conventional cassava breeding cycle spans at least four years due to the need to collect phenotypic data across four main stages of improvement: clonal evaluation trials (CET), preliminary yield trials (PYT), advanced yield trials (AYT), and uniform yield trials (UYT) (Oliveira et al., 2012). In practice, a clone typically only returns to the crossing block after being evaluated in advanced yield trials (AYT), resulting in a cassava breeding cycle lasting between 4 to 6 years.

In contrast, selecting clones for agronomic validation, which occurs after completing the UYT trials, typically takes about eight years from the start of crossings (Wolfe et al., 2017). This prolonged selection cycle can be significantly shortened with the use of genomic selection. By employing GEBVs and GETGVs in population improvement, the breeding cycle can be expedited, allowing for the selection and reintroduction of promising clones into the breeding block in a shorter time frame. Furthermore, this approach accelerates the development of new cassava cultivars (performance *per se*), as the selection of genotypes with the highest potential to become cultivars can be made earlier at the seedling stage, thus reducing the time needed for agronomic validation. Integrating genomic selection into the breeding process can shorten the conventional eight-year cycle to a considerably shorter period, leading to faster advancements in the productivity and quality of new cassava varieties. This efficiency is primarily dependent on the production of propagative material for trials with replications across multiple environments and growing seasons.

Regardless of the outcomes for genotypes in breeding programs, selecting superior genotypes based on genomic values often shows less agreement with selection based on phenotypic BLUPs, especially when truncation selection is used for traits with complex inheritance. Complex traits are controlled by numerous small-effect loci scattered throughout the genome, and genomic selection accounts for all these loci simultaneously. In contrast, phenotypic selection relies solely on observed performance, which can be affected by environmental factors and gene-environment interactions (Crossa et al., 2010; Bhat et al., 2016). The low heritability of these complex traits and their sensitivity to environmental variations can cause significant variability in the accuracy of phenotypic selection, particularly under non-uniform conditions (de los Campos et al., 2009). On the other hand, genomic selection faces accuracy challenges due to linkage disequilibrium (LD) between SNP markers and QTLs, as well as the effective population size. In populations with low LD, large populations can reduce the accuracy of genomic models (Cruz et al., 2013). The density and number of SNP markers are also critical factors, with higher marker density potentially improving the efficiency of genomic models (González-Camacho et al., 2012).

de Andrade et al. (2019) also reported a low Kappa coefficient (0.40) when analyzing the agreement in ranking clones based on genomic values versus phenotypic BLUPs, using different genomic prediction methods (BayesB, G-BLUP, RKHS, RR-BLUP, and BLASSO). In contrast, there was a high level of agreement (Kappa coefficient ranging from 0.76 to 1.0) in selecting cassava genotypes ranked based on GEBVs versus GETGVs.

In this study, cassava clones were selected based on GEBVs to advance population improvement, aiming to gradually raise the population mean for specific traits (Chen et al., 2023). GEBVs estimate the genetic value of an individual as a parent, indicating its potential to transmit favorable traits to the next generation (Crossa et al., 2017). Conversely, ranking based on GETGVs is used for selecting clones for agronomic validation in different environments experiencing drought stress, focusing on improving the individual performance of genotypes by considering both additive and non-additive effects (such as dominance and epistasis) (Chen et al., 2019).

Although higher selection intensities (e.g., 10%) result in greater selection differentials compared to the mean of the original population, the reduction in gains when increasing the selection intensity from 10% to 15% is relatively small, regardless of whether the selection is based on GEBVs or GETGVs. Bernardo et al. (2006) noted that while higher selection intensity can lead to increased genetic gains, it is generally more advisable to determine the number of parental lines based on the breeding program’s long-term or short-term goals, considering the specific characteristics of each species. In cassava breeding, crossing blocks with specific objectives typically consist of around 50 genotypes. With selection intensities of 10% and 15%, 42 and 62 genotypes would be selected, respectively, to form crossing blocks and conduct agronomic evaluations. Considering the lack of synchronization in flowering and the fact that not all cassava clones flower within a production cycle, selecting 62 clones is a reasonable number to start a genomic selection program focused on enhancing drought tolerance.

Selecting parents for crossing is a critical component of plant breeding programs. Selection intensities ranging from 5% to 30% are commonly applied, with 10% frequently used to balance selection intensity and genetic diversity (Falconer and Mackay, 1996). For instance, Das et al. (2021) used a 5% selection intensity to identify drought-tolerant maize individuals, while Zhang et al. (2017) varied selection intensities from 5% to 50% across different selection cycles to estimate genetic gains and investigate diversity in maize populations. In cassava, Sampaio Filho et al. (2023) applied a 30% selection intensity to determine gains in stability and performance, whereas Andrade et al. (2022) evaluated selection intensities ranging from 5% to 30% for selecting clones and parental lines for breeding blocks.

The success of hybridization in breeding programs depends on the appropriate selection of germplasm to be used as parents (Oliveira et al., 2017). Superior parents with desirable alleles and minimal undesirable genetic load can be identified and incorporated into breeding programs to develop cultivars with preferred allele combinations (Varshney et al., 2021). In our study, using a 15% selection intensity (62 clones), selection differentials based on GEBVs ranged from 0.76% for Stem.D to 13.50% for FRY, while gains based on GETGVs ranged from 0.81% to 13.62% for the same traits. Regardless of the genomic selection parameter (GEBV or GETGV), traits with the highest selection differentials (>7%) were economically significant (StY, ShY, and FRY). With the development of populations derived from crossing these parental lines, it is expected that selection indices will further enhance genetic gains for productive traits and other important physiological traits under drought stress. Andrade et al. (2022) reported that reducing the cassava breeding cycle makes genomic selection gains, on average, 12.48% and 11.92% higher than those from phenotypic selection for FRY and DRY traits, respectively. Oliveira et al. (2012) also noted that a 25% reduction in breeding cycle time resulted in relative efficiency improvements of 4.6%, 15.96%, and -7.05% for FRY, starch content, and DMC, respectively, with RR-BLUP genomic prediction.

### 4.4 Prospects for future breeding

Phenotypic selection can achieve genetic gains of around 5-10% per cycle, depending on the trait and the population structure (Carena et al., 2010). However, these gains are often limited by the long duration of breeding cycles and the lower precision of phenotypic evaluations. As a result, new selection approaches, such as genomic selection, are increasingly being integrated into breeding programs. Numerous studies have shown that genomic selection accelerates the breeding process by enabling earlier selection based on GEBVs, thus bypassing the need for full phenotypic cycles (Crossa et al., 2017). This advantage is especially valuable for crops with long generation intervals, like cassava, where traditional breeding methods are slow and resource-intensive (Wolfe et al., 2017).

By reducing the breeding cycle time, greater selection gains can be achieved per unit of time, even with less precise selection. For example, Oliveira et al. (2012) found that shortening the selection cycle by one year made genomic selection 4.6% more efficient than phenotypic selection for the FRY trait. These gains could be even more substantial (up to 73%) if the cassava selection cycle were reduced from 4-5 years to just 2 years. This reduction is critical, particularly for semi-perennial crops like cassava, which have naturally long production and breeding cycles (Varshney et al., 2021). Specifically, for drought tolerance in cassava, using GEBVs and GETGVs not only shortens the breeding cycle—potentially enabling annual cycles—but also optimizes resources by reducing both the costs and time associated with intensive drought stress phenotyping. Indeed, Bernardo (2008) suggested that while the gains per cycle may not necessarily be higher with marker-based selection, markers improve the efficiency of gains per unit of cost and time.

Reducing the time required to produce progeny can significantly enhance genetic gains, as shortening the reproduction cycle has a greater impact than increasing heritability or selection intensity (Cobb et al., 2019; Santos et al., 2024). In conventional cassava breeding programs, selection cycles using only phenotypic BLUPs can take 4 to 6 years due to slow vegetative propagation and the need for phenotyping genotypes in multiple environments (Mohammadi, 2018). In contrast, genomic selection can shorten this cycle to about one year, depending on the program’s ability to generate new progeny and the available genotyping infrastructure.

The decreasing costs of genotyping offer substantial long-term benefits for selecting drought tolerance compared to the intensive and costly phenotyping required for BLUP-based programs. Genomic selection allows for early selection of young plants before they reach reproductive maturity, optimizing resource use and enabling more selection cycles within a shorter timeframe. This approach increases the likelihood of accumulating favorable alleles for drought tolerance and enables a quick response to environmental and genetic changes (Costa-Neto et al., 2021). By accelerating the development of new varieties suited for challenging cultivation conditions, genomic selection also prepares breeding programs to tackle future climate challenges in increasingly demanding tropical agricultural systems that require rapid adaptation (Heffner et al., 2009; Varshney et al., 2021).

Our results suggest that genomic selection can be just as effective as traditional phenotypic selection in identifying drought-tolerant genotypes. This is crucial for breeding programs focused on developing resilient crops in response to climate change and water scarcity. Therefore, incorporating genomic selection into breeding programs can significantly expedite the development of drought-tolerant varieties, contributing to agricultural sustainability in a context of limited water resources.

### 4.5 Conclusion

According to our results, the heritability estimates for most agronomic traits were lower than those reported in the literature for cassava trials conducted under normal conditions. This suggests that water stress may have affected plant growth and physiological development, creating challenges for selecting genotypes with drought tolerance. Although the predictive abilities of GEBVs and GETGVs under water stress were low (ranging from 0.154 to 0.371), traits such as FRY, Plant.Height, Root.Di, Root.Le, ShY, and Stem.D exhibited the highest predictive abilities (>0.30). The selection differentials based on a 15% selection intensity (62 genotypes) were higher for economically significant traits like StY, ShY, and FRY, both for population improvement (using GEBVs) and for evaluating the genotype’s performance per se (using GETGVs).

Integrating genomic selection can enhance the selection of cassava genotypes under water stress by predicting the genetic value of individuals for key agronomic traits related to drought tolerance at an early stage. In conventional cassava breeding programs, selection cycles using phenotypic BLUPs can last 4 to 6 years, whereas genomic selection can feasibly reduce this cycle to about 2 years. Shortening the breeding cycle has a greater impact on genetic gain than increasing heritability or selection intensity. Additionally, decreasing genotyping costs offer significant long-term benefits compared to the intensive and costly phenotyping required for mitigating the impacts of water stress.

## Data Availability

The datasets presented in this study can be found in online repositories. The names of the repository/repositories and accession number(s) can be found below: https://figshare.com/, 10.6084/m9.figshare.26781421.
