# Supplementary material for: Optimizing drought tolerance in cassava through genomic selection
Source: Front Plant Sci. 2024 Dec 16;15:1483340. doi: 10.3389/fpls.2024.1483340 (PMC11683140; doi:10.3389/fpls.2024.1483340)
Supplement: Supplementary file 1 [file DataSheet1.docx]

Optimizing drought tolerance in cassava through genomic selection

**Supplement**


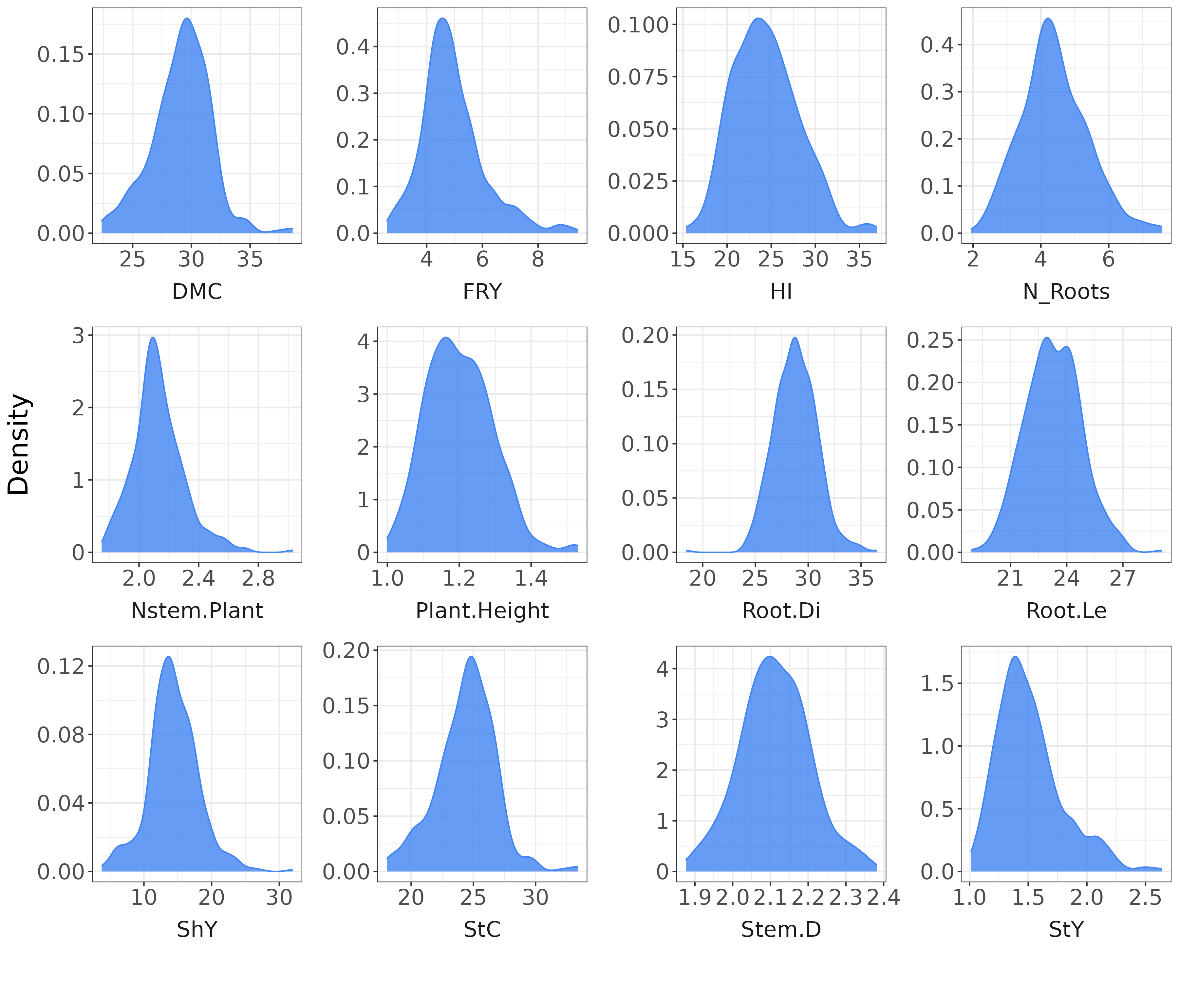


**Figure S1.** Distribution of the best linear unbiased prediction (BLUP) added to the overall mean for various traits evaluated under water deficit conditions. Traits include dry matter content (DMC), fresh root yield (FRY), harvest index (HI), number of roots per plant (N_Roots), number of stems per plant (Nstem.Plant), plant height (Plant.Height), root diameter (Root.Di), root length (Root.Le), shoot yield (ShY), starch content (StC), stem diameter (Stem.D), and starch yield (StY).


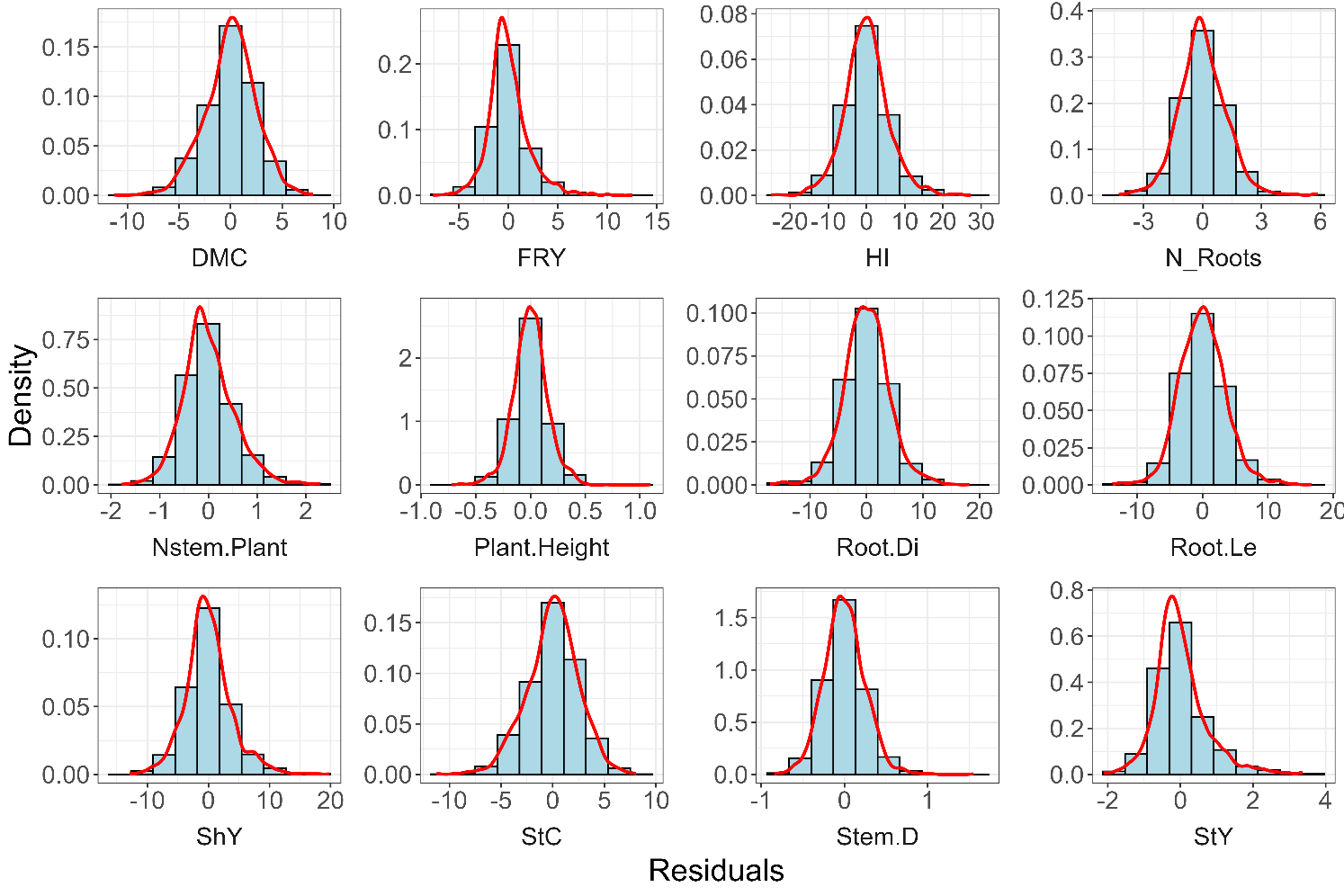


**Figure S2.** Distribution of residuals for various traits evaluated under water deficit conditions, including dry matter content (DMC), fresh root yield (FRY), harvest index (HI), number of roots per plant (N_Roots), number of stems per plant (Nstem.Plant), plant height (Plant.Height), root diameter (Root.Di), root length (Root.Le), shoot yield (ShY), starch content (StC), stem diameter (Stem.D), and starch yield (StY).


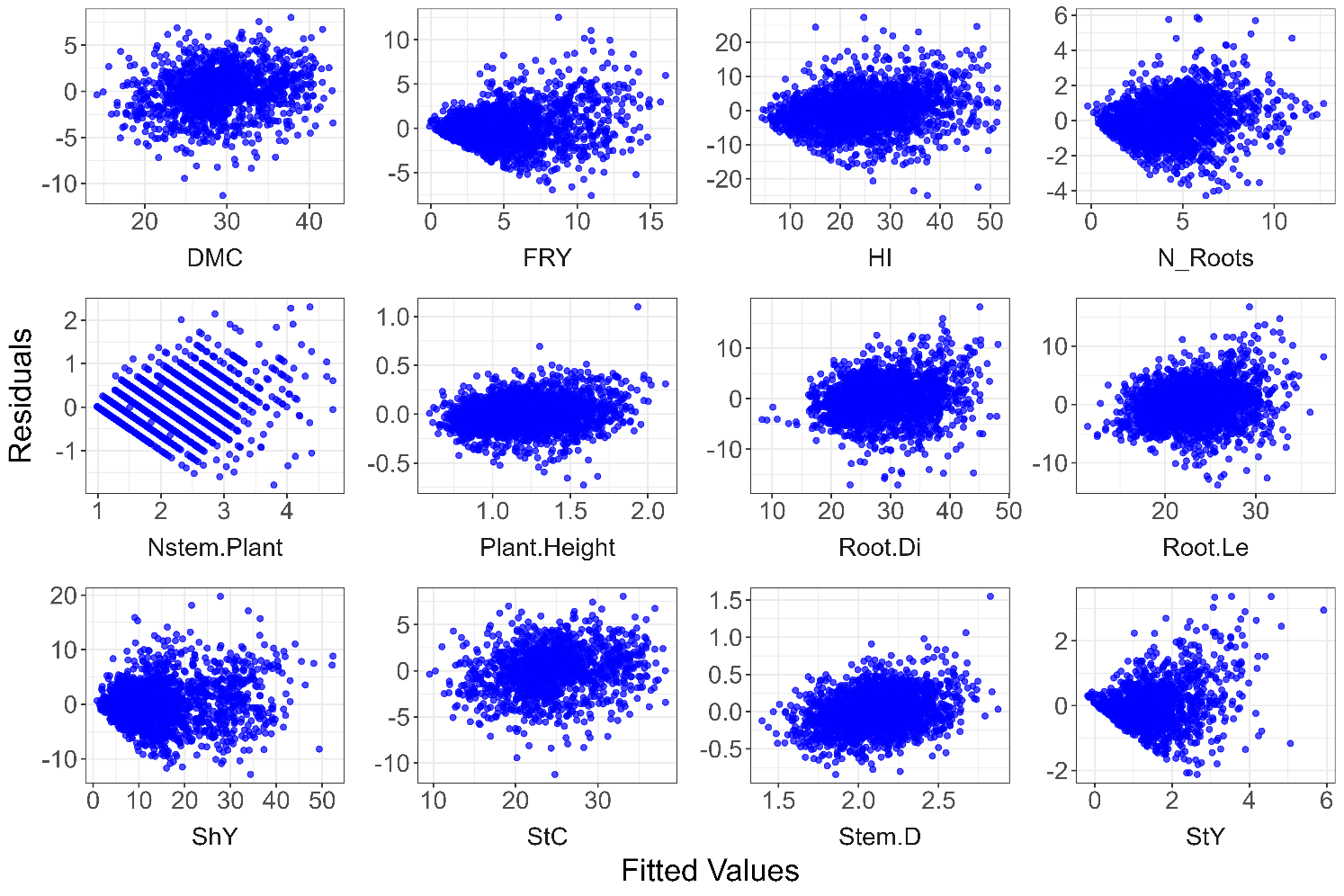


**Figure S3.** The scatterplots of the relationship between the residuals and the fitted values for various traits evaluated under water deficit conditions. Traits include dry matter content (DMC), fresh root yield (FRY), harvest index (HI), number of roots per plant (N_Roots), number of stems per plant (Nstem.Plant), plant height (Plant.Height), root diameter (Root.Di), root length (Root.Le), shoot yield (ShY), starch content (StC), stem diameter (Stem.D), and starch yield (StY).

**Table S1.** Climate data for two locations in Petrolina, Brazil, over a five-year period. The data is separated by semester (January-June and July-December) and includes the following variables: evaporation (Eva), daylight hours (DH), total rainfall (Rain), maximum temperature (MaxT), average temperature (AvT), minimum temperature (MinT), maximum humidity (MaxH), minimum humidity (MinH), and average wind speed (AvWind).

| Year | Semester | Eva | DH | Rain | MaxT | AvT | MinT | MaxH | MinH | AvWind |
| --- | --- | --- | --- | --- | --- | --- | --- | --- | --- | --- |
| 2016 | 1-6 | 10.61 | 8.45 | 1.78 | 32.95 | 27.85 | 23.30 | 65.64 | 55.34 | 2.70 |
| 2016 | 7-12 | 13.77 | 9.55 | 0.16 | 33.73 | 28.05 | 22.65 | 58.46 | 47.90 | 3.16 |
| 2017 | 1-6 | 12.41 | 8.51 | 0.58 | 33.51 | 28.48 | 23.78 | 60.44 | 50.21 | 2.97 |
| 2017 | 7-12 | 13.03 | 8.69 | 0.23 | 32.30 | 26.99 | 22.09 | 55.84 | 44.89 | 3.28 |
| 2018 | 1-6 | 9.98 | 7.92 | 1.46 | 32.40 | 27.39 | 23.12 | 62.38 | 50.77 | 2.78 |
| 2018 | 7-12 | 12.98 | 9.20 | 0.32 | 33.13 | 27.72 | 22.60 | 54.04 | 43.56 | 3.02 |
| 2019 | 1-6 | 10.84 | 8.50 | 1.14 | 33.57 | 28.31 | 23.89 | 61.07 | 48.66 | 2.76 |
| 2019 | 7-12 | 13.90 | 9.23 | 0.20 | 33.72 | 27.91 | 22.60 | 53.57 | 43.01 | 3.24 |
| 2020 | 1-6 | 8.19 | 6.87 | 1.93 | 32.20 | 27.24 | 23.48 | 70.01 | 59.37 | 2.56 |
| 2020 | 7-12 | 12.38 | 8.85 | 0.96 | 32.80 | 27.07 | 21.95 | 59.94 | 47.13 | 3.02 |

**Table S2**. Deviance analysis for various traits evaluated in cassava clones under water deficit conditions.

| Trait^1^ | Model | DF^2^ | AIC | BIC | loLik | Chisq | ChiDf | PrChisq |  |
| --- | --- | --- | --- | --- | --- | --- | --- | --- | --- |
| N_Roots | Full model | 66 | 441.82 | 553.98 | -200.91 |  |  |  |  |
|  | Clone:Year | 65 | 510.88 | 623.04 | -235.44 | 69.07 | 1.00 | 0.00 | *** |
|  | Year | 65 | 441.82 | 553.98 | -200.91 | 0.00 | 1.00 | 1.00 |  |
|  | Clone | 65 | 501.17 | 613.32 | -230.58 | 59.35 | 1.00 | 0.00 | *** |
|  | Row | 65 | 441.82 | 553.98 | -200.91 | 0.00 | 1.00 | 1.00 |  |
|  | Col | 65 | 453.85 | 566.01 | -206.93 | 12.03 | 1.00 | 0.00 | *** |
| FRY | Full model | 66 | 949.00 | 1061.03 | -454.50 |  |  |  |  |
|  | Clone:Year | 65 | 1096.46 | 1208.49 | -528.23 | 147.46 | 1.00 | 0.00 | *** |
|  | Year | 65 | 949.00 | 1061.03 | -454.50 | 0.00 | 1.00 | 1.00 |  |
|  | Clone | 65 | 984.08 | 1096.10 | -472.04 | 35.07 | 1.00 | 0.00 | *** |
|  | Row | 65 | 957.08 | 1069.10 | -458.54 | 8.07 | 1.00 | 0.00 | ** |
|  | Col | 65 | 970.33 | 1082.36 | -465.17 | 21.33 | 1.00 | 0.00 | *** |
| ShY | Full model | 66 | -281.89 | -168.66 | 160.94 |  |  |  |  |
|  | Clone:Year | 65 | -208.27 | -95.04 | 124.13 | 73.62 | 1.00 | 0.00 | *** |
|  | Year | 65 | -281.89 | -168.66 | 160.94 | 0.00 | 1.00 | 1.00 |  |
|  | Clone | 65 | -233.80 | -120.57 | 136.90 | 48.09 | 1.00 | 0.00 | *** |
|  | Row | 65 | -257.55 | -144.32 | 148.78 | 24.34 | 1.00 | 0.00 | *** |
|  | Col | 65 | -272.28 | -159.05 | 156.14 | 9.60 | 1.00 | 0.00 | ** |
| DMC | Full model | 54 | 198.14 | 281.62 | -83.07 |  |  |  |  |
|  | Clone:Year | 53 | 224.32 | 307.79 | -96.16 | 26.17 | 1.00 | 0.00 | *** |
|  | Year | 53 | 198.14 | 281.62 | -83.07 | 0.00 | 1.00 | 1.00 |  |
|  | Clone | 53 | 226.38 | 309.86 | -97.19 | 28.23 | 1.00 | 0.00 | *** |
|  | Row | 53 | 221.16 | 304.64 | -94.58 | 23.02 | 1.00 | 0.00 | *** |
|  | Col | 53 | 234.35 | 317.83 | -101.18 | 36.21 | 1.00 | 0.00 | *** |
| StY | Full model | 54 | 903.72 | 987.08 | -435.86 |  |  |  |  |
|  | Clone:Year | 53 | 980.51 | 1063.88 | -474.26 | 76.79 | 1.00 | 0.00 | *** |
|  | Year | 53 | 903.72 | 987.08 | -435.86 | 0.00 | 1.00 | 1.00 |  |
|  | Clone | 53 | 916.34 | 999.70 | -442.17 | 12.62 | 1.00 | 0.00 | *** |
|  | Row | 53 | 909.82 | 993.18 | -438.91 | 6.10 | 1.00 | 0.01 | * |
|  | Col | 53 | 946.19 | 1029.56 | -457.10 | 42.47 | 1.00 | 0.00 | *** |
| Plant.Height | Full model | 66 | 476.48 | 589.63 | -218.24 |  |  |  |  |
|  | Clone:Year | 65 | 524.51 | 637.66 | -242.25 | 48.03 | 1.00 | 0.00 | *** |
|  | Year | 65 | 476.48 | 589.63 | -218.24 | 0.00 | 1.00 | 1.00 |  |
|  | Clone | 65 | 506.78 | 619.93 | -233.39 | 30.30 | 1.00 | 0.00 | *** |
|  | Row | 65 | 514.99 | 628.13 | -237.49 | 38.50 | 1.00 | 0.00 | *** |
|  | Col | 65 | 510.55 | 623.70 | -235.28 | 34.07 | 1.00 | 0.00 | *** |
| HI | Full model | 66 | 852.67 | 964.63 | -406.33 |  |  |  |  |
|  | Clone:Year | 65 | 998.80 | 1110.76 | -479.40 | 146.13 | 1.00 | 0.00 | *** |
|  | Year | 65 | 852.67 | 964.63 | -406.33 | 0.00 | 1.00 | 1.00 |  |
|  | Clone | 65 | 883.43 | 995.40 | -421.72 | 30.76 | 1.00 | 0.00 | *** |
|  | Row | 65 | 859.27 | 971.24 | -409.64 | 6.61 | 1.00 | 0.01 | * |
|  | Col | 65 | 876.37 | 988.33 | -418.18 | 23.70 | 1.00 | 0.00 | *** |
| StC | Full model | 42 | 189.50 | 251.80 | -82.75 |  |  |  |  |
|  | Clone:Year | 41 | 216.76 | 279.06 | -96.38 | 27.27 | 1.00 | 0.00 | *** |
|  | Year | 41 | 189.50 | 251.80 | -82.75 | 0.00 | 1.00 | 1.00 |  |
|  | Clone | 41 | 211.01 | 273.31 | -93.50 | 21.51 | 1.00 | 0.00 | *** |
|  | Row | 41 | 214.93 | 277.22 | -95.46 | 25.43 | 1.00 | 0.00 | *** |
|  | Col | 41 | 225.39 | 287.68 | -100.69 | 35.89 | 1.00 | 0.00 | *** |
| Root.Le | Full model | 66 | 1117.54 | 1229.52 | -538.77 |  |  |  |  |
|  | Clone:Year | 65 | 1140.00 | 1251.99 | -550.00 | 22.47 | 1.00 | 0.00 | *** |
|  | Year | 65 | 1117.54 | 1229.52 | -538.77 | 0.00 | 1.00 | 1.00 |  |
|  | Clone | 65 | 1142.42 | 1254.41 | -551.21 | 24.88 | 1.00 | 0.00 | *** |
|  | Row | 65 | 1125.48 | 1237.47 | -542.74 | 7.95 | 1.00 | 0.00 | ** |
|  | Col | 65 | 1123.28 | 1235.27 | -541.64 | 5.74 | 1.00 | 0.02 | * |
| Root.Di | Full model | 66 | 664.19 | 776.18 | -312.09 |  |  |  |  |
|  | Clone:Year | 65 | 723.77 | 835.76 | -341.89 | 59.58 | 1.00 | 0.00 | *** |
|  | Year | 65 | 664.19 | 776.18 | -312.09 | 0.00 | 1.00 | 1.00 |  |
|  | Clone | 65 | 693.59 | 805.58 | -326.80 | 29.40 | 1.00 | 0.00 | *** |
|  | Row | 65 | 704.32 | 816.31 | -332.16 | 40.13 | 1.00 | 0.00 | *** |
|  | Col | 65 | 672.04 | 784.03 | -316.02 | 7.85 | 1.00 | 0.01 | ** |
| Stem.D | Full model | 54 | 1572.25 | 1662.44 | -770.13 |  |  |  |  |
|  | Clone:Year | 53 | 1623.51 | 1713.69 | -795.75 | 51.25 | 1.00 | 0.00 | *** |
|  | Year | 53 | 1572.25 | 1662.44 | -770.13 | 0.00 | 1.00 | 1.00 |  |
|  | Clone | 53 | 1589.92 | 1680.11 | -778.96 | 17.67 | 1.00 | 0.00 | *** |
|  | Row | 53 | 1579.60 | 1669.78 | -773.80 | 7.34 | 1.00 | 0.01 | ** |
|  | Col | 53 | 1593.90 | 1684.08 | -780.95 | 21.64 | 1.00 | 0.00 | *** |
| Nstem.Plant | Full model | 42 | 635.66 | 698.97 | -305.83 |  |  |  |  |
|  | Clone:Year | 41 | 695.98 | 759.30 | -335.99 | 60.33 | 1.00 | 0.00 | *** |
|  | Year | 41 | 635.66 | 698.97 | -305.83 | 0.00 | 1.00 | 1.00 |  |
|  | Clone | 41 | 646.05 | 709.37 | -311.02 | 10.39 | 1.00 | 0.00 | ** |
|  | Row | 41 | 635.66 | 698.97 | -305.83 | 0.00 | 1.00 | 1.00 |  |
|  | Col | 41 | 635.66 | 698.97 | -305.83 | 0.00 | 1.00 | 1.00 |  |

^1^: dry matter content (DMC), fresh root yield (FRY), harvest index (HI), number of roots per plant (N_Roots), number of stems per plant (Nstem.Plant), plant height (Plant.Height), root diameter (Root.Di), root length (Root.Le), shoot yield (ShY), starch content (StC), stem diameter (Stem.D), and starch yield (StY).

^2^: degree of freedom

**Table S3**. Cohen’s Kappa (k) of coincidence in selecting cassava clones based on its GEBVs and BLUPs considering different intensity selection (10% to 30%—SP) for several traits, such : Starch Content (StC), Root Length (Root.Le), Stem Diameter (Stem.D), Root Diameter (Root.Di), Harvest Index (HI), Dry Matter Content (DMC), Plant Height (Plant.Height), Number of Stems per Plant (Nstem.Plant), Number of Roots per Plant (N_Roots), Shoot Yield (ShY), Starch Yield (StY) and Fresh Root Yiel (FRY).

| Trait | SI | k_GEBV_BLUP | k_GETGV_BLUP | k_GETGV_GEBV | k_sel_GEBV_BLUP_BLUP | k_sel_GETGV_BLUP_BLUP | k_sel_GETGV_GEBV_BLUP |
| --- | --- | --- | --- | --- | --- | --- | --- |
| N_Roots | 10 | 0.059 | 0.115 | 0.806 | 0.585 | 0.585 | 0.115 |
| N_Roots | 15 | 0.104 | 0.065 | 0.825 | 0.513 | 0.513 | 0.065 |
| N_Roots | 20 | 0.080 | 0.080 | 0.831 | 0.524 | 0.509 | 0.080 |
| N_Roots | 25 | 0.071 | 0.123 | 0.882 | 0.516 | 0.529 | 0.110 |
| N_Roots | 30 | 0.090 | 0.102 | 0.860 | 0.522 | 0.533 | 0.113 |
| FRY | 10 | 0.004 | 0.087 | 0.862 | 0.585 | 0.723 | 0.087 |
| FRY | 15 | 0.250 | 0.193 | 0.808 | 0.673 | 0.712 | 0.250 |
| FRY | 20 | 0.218 | 0.218 | 0.877 | 0.601 | 0.632 | 0.233 |
| FRY | 25 | 0.189 | 0.189 | 0.882 | 0.607 | 0.647 | 0.189 |
| FRY | 30 | 0.280 | 0.257 | 0.872 | 0.617 | 0.675 | 0.280 |
| ShY | 10 | -0.028 | -0.001 | 0.811 | 0.567 | 0.567 | -0.001 |
| ShY | 15 | 0.020 | 0.097 | 0.827 | 0.577 | 0.558 | 0.058 |
| ShY | 20 | 0.149 | 0.179 | 0.878 | 0.529 | 0.498 | 0.164 |
| ShY | 25 | 0.238 | 0.225 | 0.845 | 0.561 | 0.509 | 0.238 |
| ShY | 30 | 0.284 | 0.214 | 0.873 | 0.538 | 0.515 | 0.237 |
| DMC | 10 | 0.024 | 0.024 | 1.000 | 0.512 | 0.512 | 0.024 |
| DMC | 15 | 0.095 | 0.095 | 1.000 | 0.511 | 0.511 | 0.095 |
| DMC | 20 | 0.147 | 0.147 | 1.000 | 0.526 | 0.526 | 0.147 |
| DMC | 25 | 0.139 | 0.139 | 1.000 | 0.554 | 0.554 | 0.139 |
| DMC | 30 | 0.099 | 0.099 | 1.000 | 0.529 | 0.529 | 0.099 |
| StY | 10 | -0.006 | -0.006 | 0.856 | 0.605 | 0.569 | -0.006 |
| StY | 15 | 0.120 | 0.120 | 0.902 | 0.609 | 0.633 | 0.144 |
| StY | 20 | 0.250 | 0.212 | 0.865 | 0.654 | 0.673 | 0.212 |
| StY | 25 | 0.282 | 0.251 | 0.920 | 0.713 | 0.729 | 0.251 |
| StY | 30 | 0.288 | 0.302 | 0.874 | 0.707 | 0.735 | 0.316 |
| Plant.Height | 10 | 0.188 | 0.188 | 0.865 | 0.567 | 0.594 | 0.188 |
| Plant.Height | 15 | 0.193 | 0.193 | 0.846 | 0.520 | 0.596 | 0.193 |
| Plant.Height | 20 | 0.255 | 0.255 | 0.924 | 0.544 | 0.605 | 0.286 |
| Plant.Height | 25 | 0.290 | 0.264 | 0.897 | 0.574 | 0.587 | 0.277 |
| Plant.Height | 30 | 0.330 | 0.295 | 0.896 | 0.642 | 0.619 | 0.307 |
| HI | 10 | -0.024 | -0.024 | 0.806 | 0.502 | 0.502 | -0.024 |
| HI | 15 | 0.078 | 0.058 | 0.865 | 0.481 | 0.500 | 0.058 |
| HI | 20 | 0.141 | 0.126 | 0.908 | 0.494 | 0.509 | 0.126 |
| HI | 25 | 0.136 | 0.149 | 0.895 | 0.516 | 0.542 | 0.136 |
| HI | 30 | 0.152 | 0.199 | 0.907 | 0.547 | 0.547 | 0.176 |
| StC | 10 | 0.024 | 0.024 | 1.000 | 0.512 | 0.512 | 0.024 |
| StC | 15 | 0.071 | 0.071 | 1.000 | 0.487 | 0.487 | 0.071 |
| StC | 20 | 0.147 | 0.147 | 1.000 | 0.526 | 0.526 | 0.147 |
| StC | 25 | 0.139 | 0.139 | 1.000 | 0.538 | 0.538 | 0.139 |
| StC | 30 | 0.127 | 0.127 | 1.000 | 0.543 | 0.543 | 0.127 |
| Root.Le | 10 | 0.142 | 0.142 | 0.862 | 0.557 | 0.613 | 0.115 |
| Root.Le | 15 | 0.299 | 0.240 | 0.844 | 0.552 | 0.552 | 0.299 |
| Root.Le | 20 | 0.340 | 0.356 | 0.847 | 0.540 | 0.570 | 0.325 |
| Root.Le | 25 | 0.320 | 0.333 | 0.895 | 0.542 | 0.581 | 0.320 |
| Root.Le | 30 | 0.347 | 0.300 | 0.872 | 0.592 | 0.603 | 0.335 |
| Root.Di | 10 | 0.115 | 0.115 | 0.972 | 0.530 | 0.530 | 0.115 |
| Root.Di | 15 | 0.143 | 0.104 | 0.961 | 0.532 | 0.532 | 0.123 |
| Root.Di | 20 | 0.233 | 0.233 | 0.969 | 0.570 | 0.555 | 0.233 |
| Root.Di | 25 | 0.254 | 0.267 | 0.961 | 0.568 | 0.555 | 0.254 |
| Root.Di | 30 | 0.253 | 0.242 | 0.965 | 0.592 | 0.545 | 0.242 |
| Stem.D | 10 | 0.161 | 0.080 | 0.756 | 0.648 | 0.567 | 0.134 |
| Stem.D | 15 | 0.204 | 0.166 | 0.848 | 0.621 | 0.602 | 0.204 |
| Stem.D | 20 | 0.270 | 0.225 | 0.802 | 0.620 | 0.635 | 0.270 |
| Stem.D | 25 | 0.303 | 0.290 | 0.845 | 0.703 | 0.703 | 0.303 |
| Stem.D | 30 | 0.298 | 0.298 | 0.816 | 0.781 | 0.747 | 0.310 |
| Nstem.Plant | 10 | 0.024 | 0.024 | 1.000 | 0.442 | 0.477 | 0.024 |
| Nstem.Plant | 15 | 0.136 | 0.112 | 0.952 | 0.472 | 0.496 | 0.136 |
| Nstem.Plant | 20 | 0.139 | 0.139 | 0.888 | 0.551 | 0.570 | 0.139 |
| Nstem.Plant | 25 | 0.250 | 0.234 | 0.969 | 0.578 | 0.625 | 0.250 |
| Nstem.Plant | 30 | 0.304 | 0.276 | 0.959 | 0.577 | 0.590 | 0.290 |

**Table S4**. Selection gains based on cassava genotypes selected using BLUPs, GEBVs, and GETGVs, considering selection intensities from 10% to 30% for various traits, including: Starch Content (StC), Root Length (Root.Le), Stem Diameter (Stem.D), Root Diameter (Root.Di), Harvest Index (HI), Dry Matter Content (DMC), Plant Height (Plant.Height), Number of Stems per Plant (Nstem.Plant), Number of Roots per Plant (N_Roots), Shoot Yield (ShY), Starch Yield (StY), and Fresh Root Yield (FRY).

| Trait | SI | N# selected clones | X0 | XS_GEBV | XS_GETGV | DS_GEBV (%) | DS_GETGV (%) |
| --- | --- | --- | --- | --- | --- | --- | --- |
| N_Roots | 10 | 42 | 4.293 | 4.530 | 4.551 | 5.513 | 6.005 |
| N_Roots | 15 | 62 |  | 4.488 | 4.513 | 4.552 | 5.135 |
| N_Roots | 20 | 83 |  | 4.474 | 4.491 | 4.220 | 4.612 |
| N_Roots | 25 | 104 |  | 4.456 | 4.469 | 3.808 | 4.111 |
| N_Roots | 30 | 125 |  | 4.444 | 4.459 | 3.509 | 3.865 |
| FRY | 10 | 42 | 4.946 | 5.614 | 5.620 | 13.505 | 13.619 |
| FRY | 15 | 62 |  | 5.526 | 5.507 | 11.731 | 11.346 |
| FRY | 20 | 83 |  | 5.451 | 5.457 | 10.208 | 10.331 |
| FRY | 25 | 104 |  | 5.390 | 5.390 | 8.968 | 8.977 |
| FRY | 30 | 125 |  | 5.334 | 5.334 | 7.834 | 7.840 |
| ShY | 10 | 42 | 14.228 | 15.563 | 15.609 | 9.387 | 9.711 |
| ShY | 15 | 62 |  | 15.301 | 15.363 | 7.546 | 7.979 |
| ShY | 20 | 83 |  | 15.213 | 15.180 | 6.929 | 6.691 |
| ShY | 25 | 104 |  | 15.142 | 15.200 | 6.424 | 6.837 |
| ShY | 30 | 125 |  | 15.057 | 15.137 | 5.826 | 6.390 |
| DMC | 10 | 42 | 29.058 | 29.644 | 29.648 | 2.015 | 2.031 |
| DMC | 15 | 62 |  | 29.564 | 29.603 | 1.740 | 1.874 |
| DMC | 20 | 83 |  | 29.495 | 29.523 | 1.504 | 1.599 |
| DMC | 25 | 104 |  | 29.444 | 29.438 | 1.327 | 1.308 |
| DMC | 30 | 125 |  | 29.399 | 29.424 | 1.174 | 1.258 |
| StY | 10 | 42 | 1.516 | 1.682 | 1.687 | 10.937 | 11.274 |
| StY | 15 | 62 |  | 1.650 | 1.650 | 8.843 | 8.829 |
| StY | 20 | 83 |  | 1.638 | 1.635 | 8.041 | 7.805 |
| StY | 25 | 104 |  | 1.624 | 1.624 | 7.124 | 7.099 |
| StY | 30 | 125 |  | 1.616 | 1.616 | 6.587 | 6.553 |
| Plant,Height | 10 | 42 | 1.192 | 1.218 | 1.214 | 2.208 | 1.881 |
| Plant,Height | 15 | 62 |  | 1.210 | 1.209 | 1.509 | 1.444 |
| Plant,Height | 20 | 83 |  | 1.207 | 1.205 | 1.227 | 1.090 |
| Plant,Height | 25 | 104 |  | 1.204 | 1.204 | 1.057 | 1.054 |
| Plant,Height | 30 | 125 |  | 1.202 | 1.205 | 0.874 | 1.140 |
| HI | 10 | 42 | 24.556 | 26.057 | 25.986 | 6.115 | 5.826 |
| HI | 15 | 62 |  | 25.853 | 25.780 | 5.284 | 4.984 |
| HI | 20 | 83 |  | 25.704 | 25.701 | 4.675 | 4.665 |
| HI | 25 | 104 |  | 25.520 | 25.475 | 3.924 | 3.744 |
| HI | 30 | 125 |  | 25.379 | 25.332 | 3.352 | 3.161 |
| StC | 10 | 42 | 24.420 | 25.004 | 25.009 | 2.392 | 2.414 |
| StC | 15 | 62 |  | 24.922 | 24.959 | 2.058 | 2.210 |
| StC | 20 | 83 |  | 24.854 | 24.881 | 1.778 | 1.888 |
| StC | 25 | 104 |  | 24.803 | 24.797 | 1.572 | 1.544 |
| StC | 30 | 125 |  | 24.759 | 24.782 | 1.390 | 1.485 |
| Root,Le | 10 | 42 | 23.215 | 23.879 | 23.848 | 2.863 | 2.726 |
| Root,Le | 15 | 62 |  | 23.768 | 23.710 | 2.381 | 2.135 |
| Root,Le | 20 | 83 |  | 23.707 | 23.657 | 2.119 | 1.904 |
| Root,Le | 25 | 104 |  | 23.642 | 23.609 | 1.839 | 1.700 |
| Root,Le | 30 | 125 |  | 23.572 | 23.607 | 1.541 | 1.690 |
| Root,Di | 10 | 42 | 28.879 | 29.681 | 29.644 | 2.777 | 2.651 |
| Root,Di | 15 | 62 |  | 29.586 | 29.573 | 2.450 | 2.405 |
| Root,Di | 20 | 83 |  | 29.519 | 29.516 | 2.219 | 2.209 |
| Root,Di | 25 | 104 |  | 29.451 | 29.443 | 1.983 | 1.955 |
| Root,Di | 30 | 125 |  | 29.384 | 29.368 | 1.751 | 1.695 |
| Stem,D | 10 | 42 | 2.112 | 2.136 | 2.135 | 1.118 | 1.065 |
| Stem,D | 15 | 62 |  | 2.132 | 2.132 | 0.901 | 0.907 |
| Stem,D | 20 | 83 |  | 2.131 | 2.130 | 0.862 | 0.810 |
| Stem,D | 25 | 104 |  | 2.129 | 2.130 | 0.802 | 0.822 |
| Stem,D | 30 | 125 |  | 2.129 | 2.130 | 0.760 | 0.811 |
| Nstem,Plant | 10 | 42 | 2.131 | 2.156 | 2.160 | 1.169 | 1.365 |
| Nstem,Plant | 15 | 62 |  | 2.153 | 2.154 | 1.014 | 1.075 |
| Nstem,Plant | 20 | 83 |  | 2.150 | 2.151 | 0.877 | 0.931 |
| Nstem,Plant | 25 | 104 |  | 2.152 | 2.149 | 0.983 | 0.859 |
| Nstem,Plant | 30 | 125 |  | 2.151 | 2.151 | 0.961 | 0.947 |
